# Factors determining the overlap between recipients of the first and second dose of measles vaccine in nineteen surveys

**DOI:** 10.1038/s41598-025-10678-8

**Published:** 2025-08-21

**Authors:** Timos Papadopoulos, Mark Jit, Matthew J. Ferrari, Emilia Vynnycky

**Affiliations:** 1 Statistics, Modelling and Economics Department, United Kingdom Health Security Agency, 61 Colindale Avenue, London, NW9 5EQ UK; 2 Department of Global and Environmental Health, New York University, 708 Broadway, New York, NY 10003 USA; 3 Center for Infectious Disease Dynamics, Department of Biology, The Pennsylvania State University, University Park, Pennsylvania, PA 16802 USA; 4 Department of Infectious Disease Epidemiology, London School of Hygiene & Tropical Medicine, Keppel Street, London, WC1E 7HT UK

**Keywords:** Health policy, Epidemiology

## Abstract

Many countries schedule a second dose of measles-containing vaccine (MCV2) for children in their second year of life. The correlation between recipients of the first dose of measles-containing vaccine (MCV1) and MCV2 is poorly understood but is important for estimating population levels of measles immunity and for meeting elimination targets. Using data from 19 surveys from Demographic and Health Surveys (DHS) we computed the percentage of MCV1 recipients with subsequent MCV2 and of MCV2 recipients with previous MCV1. All countries included in our study recommended MCV1 in the first year of life and MCV2 in the second year of life. For 2 surveys we computed the variation of those percentages over the country’s geographical regions. We computed adjusted odds ratios for the association of this percentage with age, sex, residency, mother’s education, wealth and birth order. For most of the surveys, over 50% of MCV1 recipients received MCV2, but there was more than 30% MCV1 to MCV2 dropout in more than half of the surveys. The percentage of MCV1 recipients with MCV2 was statistically significantly higher if they received MCV1 below age 12 months and the percentage increased with increasing education status of the mother and higher income levels. A small number of MCV2 recipients were not found to have received MCV1, despite marked on record as having received MCV2 implies having previously received MCV1 (by definition of the survey data collection methodology). Our analyses have highlighted important shortfalls by age, country, mother’s education and income status in the proportion of MCV1 recipients who subsequently receive MCV2. Targeting those differentials is essential for achieving the goals of measles elimination.

**Supplementary Information:**

The online version contains supplementary material available at 10.1038/s41598-025-10678-8.

## Introduction

WHO currently recommends that, in addition to a first routine dose of measles containing vaccination (MCV1), all countries should include a second routine dose (MCV2) in their childhood vaccination schedule^1^. Providing a second opportunity for vaccination is important as it helps to immunize children who either failed to develop immunological protection following their first dose or who missed the first dose altogether. The recommendation for the timing of the two doses depends on the level of measles transmission in each country and MCV1 is generally recommended before or at the end of the first year of life and MCV2 in the second year of life^1,2^.

An understanding of the proportion of MCV1 recipients who then receive the second measles vaccine dose is important for meeting targets of the Measles and Rubella Strategic Framework 2021–2030^3^, which has been developed following the Immunization Agenda 2030 (IA2030) structure^4^. As part of this framework, all six WHO regions have established or expressed a commitment to achieving regional elimination of both measles and rubella^3^. In the period 2012–2022 rubella elimination has been achieved in one WHO region (Americas in 2015) and 98 countries^5^. In contrast, fewer countries (84 in year 2023) have reported elimination and no entire WHO region has achieved and maintained elimination of measles^6^. In common with the target coverage for all essential vaccines given in childhood and adolescence, the set target is for 90% global coverage with MCV2 (defined as the proportion of children who have received 2nd dose of measles-containing vaccine, according to the nationally recommended schedule) by 2030^7^. MCV2 coverage is used as an indicator of progress towards achieving SDG3 (p.6 in^8^) and measles vaccination coverage is used to indicate the strength of immunization programmes in the IA2030^4^.

Despite its importance in evaluating the progress towards those targets, the proportion of MCV1 recipients who then receive the second dose as well as the related dropout, i.e. the proportion of MCV1 recipients who do not go on to receive MCV2, are poorly understood. An understanding of the proportion of recipients of MCV1 who then receive MCV2 is important for the levels of immunity in the population and therefore for predicting the impact of vaccination programs^9^. For example, modelling studies sometimes assume that vaccine doses are independent, so that children are as likely to receive the dose scheduled at the time of the second dose as they are to receive the first dose^10,11^. Such an assumption may lead to an overestimate in the proportion of children that are protected by vaccination and, consequently, an overestimate in the impact of vaccination, if recipients of both doses are to a great degree identical^9,12^. These questions regarding correlation between doses are also relevant for predicting the impact of rubella vaccination, as countries considering introducing rubella vaccination into their routine schedule are recommended to provide it as part of the measles schedule, as a combined measles-rubella vaccine^13^.

To date, studies have typically calculated the proportion of children that have received both doses of a given vaccine (were “fully vaccinated”) or the proportion of children that have had “zero doses” of measles vaccination by a given age. For example, Portnoy et al.^14^ explored the effectiveness of measles vaccination campaigns (supplementary immunization activities – SIAs) in reaching children who had not yet received measles vaccination at all (“zero-dose” children), considering the proportion of zero-dose children reached by SIAs in different countries and its variation with household wealth and other factors. That study also examined the impact of including or excluding different types of immunization information in the analysis, namely of information obtained from vaccination cards or, in the absence of that, of information provided verbally by the mother about the child’s vaccination history. They find that such different interpretations of the DHS records can lead to large differences in the estimate of the proportion of measles ‘zero-dose’ children who are reached by measles vaccination campaigns. In a wider systematic survey on the reliability of different types of documented evidence to determine a child’s vaccination history, including vaccination cards, maternal recall, health facility sources and others, Dansereau et al.^15^ found that vaccination cards and maternal recall provided relatively consistent vaccination histories in some settings but were in poor agreement with other types of evidence.

To our knowledge, the existing evidence on the proportion or the characteristics of those who have received MCV1 and have subsequently received a second dose is limited. Masresha et al.^16^ use the difference of MCV1 to MCV2 coverage relative to MCV1 coverage to estimate the MCV1 to MCV2 dropout. Hailu et al.^17^ examine the proportion of children who received the Pentavalent vaccine who then received MCV1. In a more recent study, Adugna et al.^18^ examine the MCV1 to MCV2 dropout in one region of Ethiopia and its association with a large number of sociodemographic indicators and health access characteristics.

Other studies on vaccination coverage^19–24^ have also found that the vaccination coverage for a number of different vaccines varied with factors, such as socio-economic status, geographical characteristics of residence and the mother’s education. Since these characteristics vary between countries and settings, the overlap between recipients of both doses is also likely to be country-specific.

In this study, we use data from 19 surveys of the Demographic Health Surveys in 17 countries to evaluate the association between recipients of vaccine doses for measles, quantified as the proportion of MCV1 recipients who then received MCV2. We also explore the association between this proportion and the demographic and socio-economic characteristics of the survey participants in each survey setting.

## Results

### Source of information and coverage of MCV1 and MCV2

**Fig. 1:**
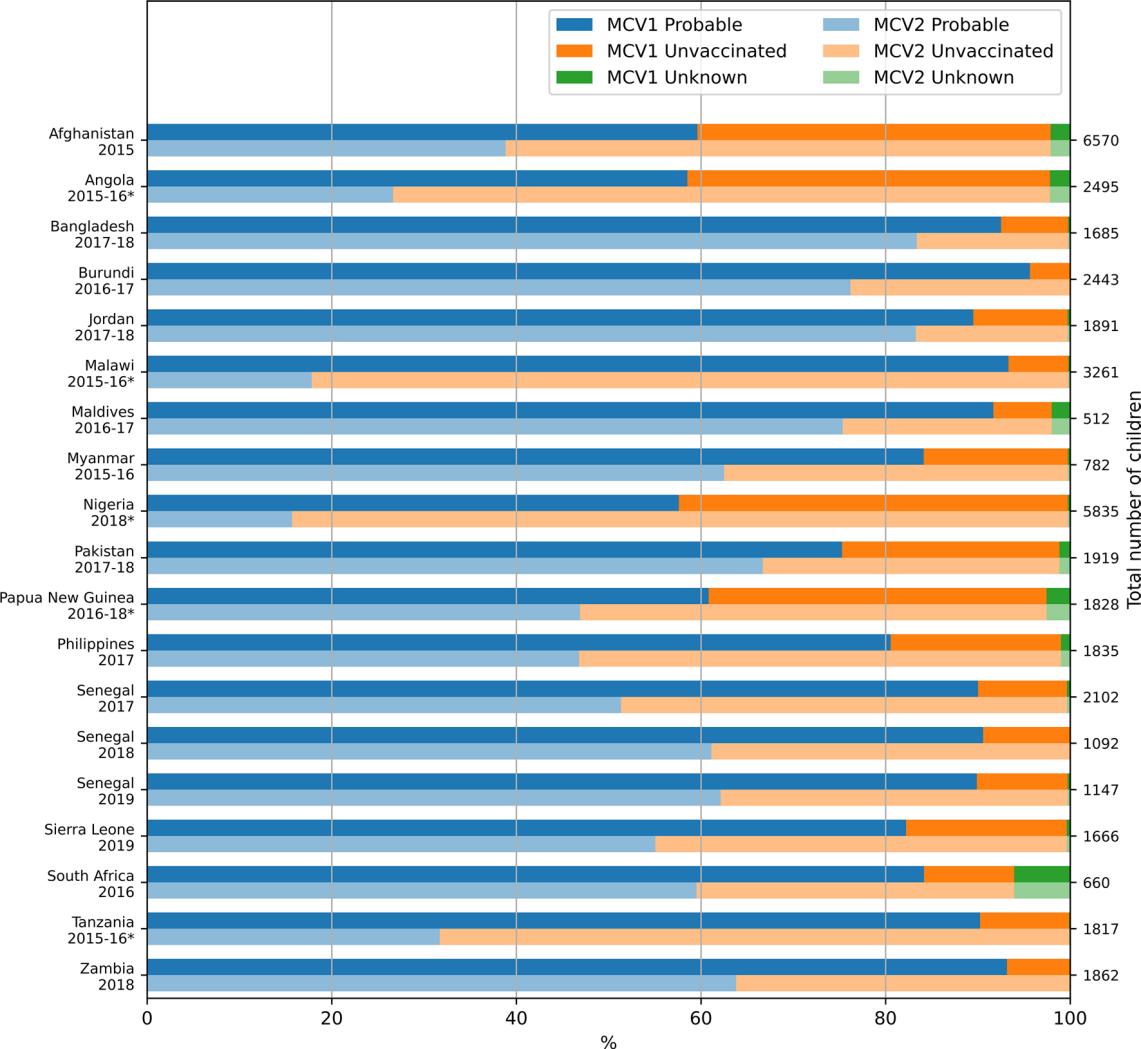
Vaccination status among 24–35 month olds in each survey. The corresponding numerical data are presented in tabular form in the supplement (Table S1). Surveys for which MCV2 was introduced less than 3 years before the survey are denoted with an asterisk.

Figure 1 and Table S1 (Supplement) summarise the MCV1 and MCV2 status among 24–35 month olds in each of the 19 DHS surveys considered in these analyses. As described in the ‘Methods’ section, we use the term ‘children with probable MCV1’ to refer to children whose vaccination card or mother’s report in the DHS data indicated that they had received MCV1. The term ‘children with probable MCV2’ is defined similarly and regardless of whether the child was reported to have had MCV1 previously. The results obtained when only children with vaccination card are considered are presented later in the results section. The percentage of children with probable MCV1 ranged from 58% in Nigeria (2018) to 96% in Burundi (2016-17). For the countries that introduced MCV2 at least three years before the corresponding survey, the percentage of children with probable MCV2 ranged from 39% in Afghanistan (2015) to 83% in Bangladesh (2017-18) and Jordan (2017-18). In countries where MCV2 was introduced less than three years before the survey, probable MCV2 ranged from 16% in Nigeria (2018) to 47% in Papua New Guinea (2016-18) where MCV2 was due to be introduced in 2020 and 2016 respectively. The percentages of children with unknown MCV2 status were identical to those for MCV1 for all surveys. This percentage was below 0.5% for 12 of the 19 studies and below 2.6% for all studies with the exception of South Africa (2016) in which it was markedly higher at 6.1%. As shown in Table S1, the probable MCV1 and probable MCV2 percentages are consistent with the corresponding WUENIC coverage estimates.

In all surveys the percentage of children with probable MCV2 was lower than the percentage of children with probable MCV1, with the difference between the two percentages ranging from 6% for Jordan (2017-18) (MCV2 introduced in 2000) to 75% in Malawi (2015-16) (MCV2 introduced in 2015). Among the countries that introduced MCV2 less than 3 years before the survey, this difference was smallest (14%) in Papua New Guinea (2016-18) and among the countries that introduced MCV2 at least 3 years before the survey it was highest (39%) in Senegal (2017). For one country (Senegal), where MCV2 was introduced in 2014, separate surveys occurred for three consecutive years (2017–2019). Here, the percentage of children with probable MCV1 was similar (90–91%) over those three years, whereas the percentage of children with probable MCV2 increased from 51% in 2017 to 62% in 2019.

As shown in Table S2 (Supplementary material), among the children who were probably vaccinated with a given dose, the proportion whose vaccination status with that dose was obtained from the vaccination card ranged from 20% in Malawi (2015-16) to 95% in Maldives (2016-17). Among the 19 surveys, more than half the children with recorded vaccination status had vaccine status information in vaccination cards in 15 and 13 surveys for MCV1 and MCV2 respectively.

**Fig. 2:**
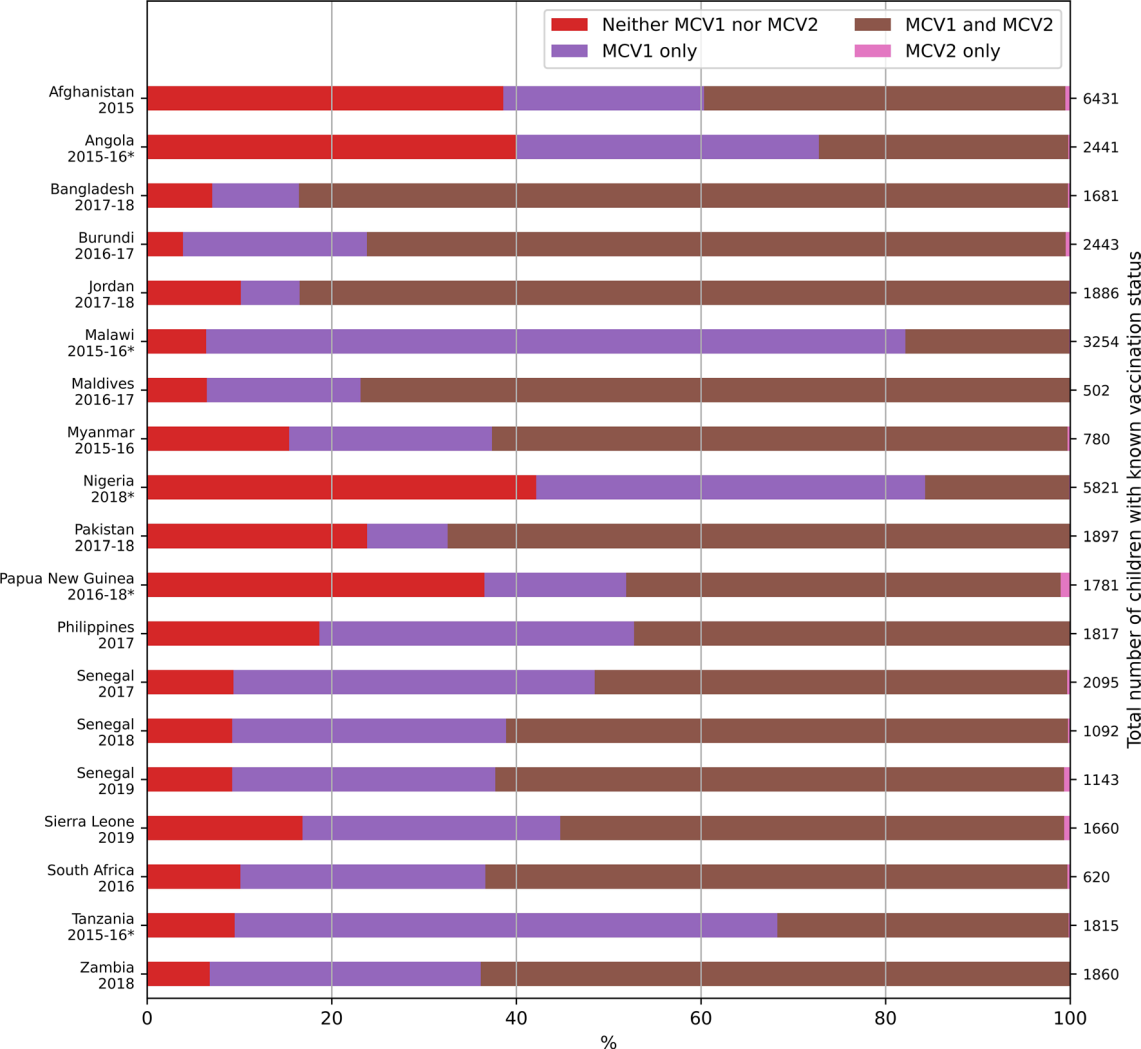
Probable MCV1 and MCV2 status among the children aged 24–35 months whose vaccination status was known. The corresponding numerical data are presented in tabular form in the supplement (Table S3). Surveys for which MCV2 was introduced less than 3 years before the survey are denoted with an asterisk.

The percentage of children who received neither MCV1 nor MCV2 ranged from 4% in Burundi (2016-17) to 42.1% in Nigeria (2018) (Figure 2 and Table S3, Supplement), and the percentage of children with probable MCV1 but not MCV2 ranged from 6% in Jordan (2017-18) to 76% in Malawi (2015-16).

**Fig. 3:**
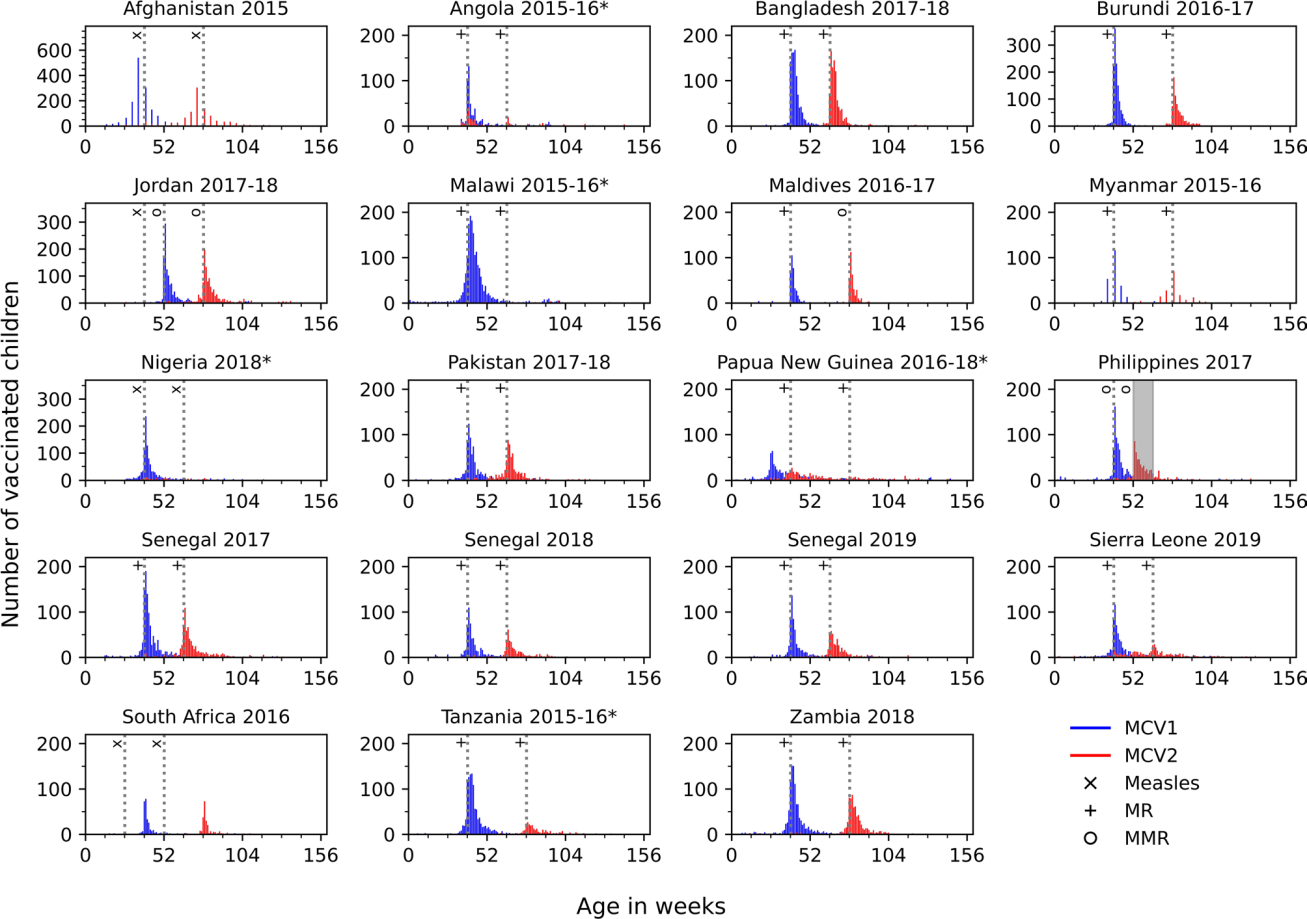
Distribution of age (in weeks) at which MCV1 and MVC2 was received. The dotted lines indicate the target age for administering MCV1 and MCV2 (MMR, MR or MEAS) as they are in the time of this publication^25^. In the Afghanistan (2015) and Myanmar (2015-6) plots the histogram bins correspond to months, as the age in weeks was not reported in these surveys. The shaded area in the Philippines (2017) plot corresponds to a dose of MMR extending to ages 12–15 months^25^. The age range extends up to the third year of age and it does not include doses scheduled in older ages (a list of scheduled doses extending to after the third year of age is included in Table S4 in the Supplement). Surveys for which MCV2 was introduced less than 3 years before the survey are denoted with an asterisk.

In Figure 3 we plot the distribution of the age, in weeks, at which MCV1 and MCV2 was given. The data for this figure are restricted to children whose measles vaccination age was marked on their vaccination card. The Afghanistan (2015) and Myanmar (2015-16) surveys allowed only the extraction of month of age at vaccination, hence the histogram bins for these surveys correspond to months of age. For the countries that introduced MCV2 at least 3 years before the survey, MCV1 was typically given below age 12 months, whilst MCV2 occurred several months later and typically after age 12 months; the actual ages at which MCV2 was received typically clustered around the corresponding scheduled age (Table S4 in the supplement, see also^25,26^ for details of how information is reported through the WHO/UNICEF Joint Reporting Process). In 3 out the 5 countries that introduced MCV2 less than 3 years before the survey (Angola (2015-16), Malawi (2015-16) and Nigeria (2018)) there are very few children with MCV2 and the age at MCV2 does not coincide with the scheduled age (15 months for Angola and Malawi and 18 months for Nigeria). In the case of Tanzania (2015-16) where MCV2 was introduced just before the survey in 2014 (see Table S1) there is a small number of children with MCV2 around the scheduled age of 18 months. In Sierra Leone (2019) for which the scheduled age for MCV2 is 15 months, the age distribution of MCV2 administration appears nearly uniform with a very small peak at the scheduled age and several cases appear of MCV2 administration up to 24 weeks before the scheduled age. In the case of Papua New Guinea (2016-18) the MCV1 and MCV2 scheduled ages listed by WHO in^25^ (9 and 18 months of age) do not coincide with the DHS data. The DHS report of the survey^27^ states that according to the National Department of Health, “the first dose of the measles and rubella vaccine should be given at or soon after the child reaches age 6–8 months, while the second dose should be given at age 9–11 months”. The same report mentions “a third dose of measles given at 18–24 months” which however “had not yet been rolled out when the fieldwork for the PNG DHS started in 2016”. This schedule coincides with the age distribution presented in the plot. Two other discrepancies with the schedule listed by WHO in^25^ are those of Jordan (2017-18) where the scheduled dose of measles vaccine at 9 months does not appear in the DHS data and of South Africa (2016) where the 6 month and 12 month scheduled ages listed by WHO were introduced after 2015. The vaccination of the children included in the South African survey followed the previous schedule of MCV1 and MCV2 at 9 and 18 months of age (pp.151-2 in^28^).

### Percentage of probable MCV1 recipients who probably received MCV2 

**Fig. 4:**
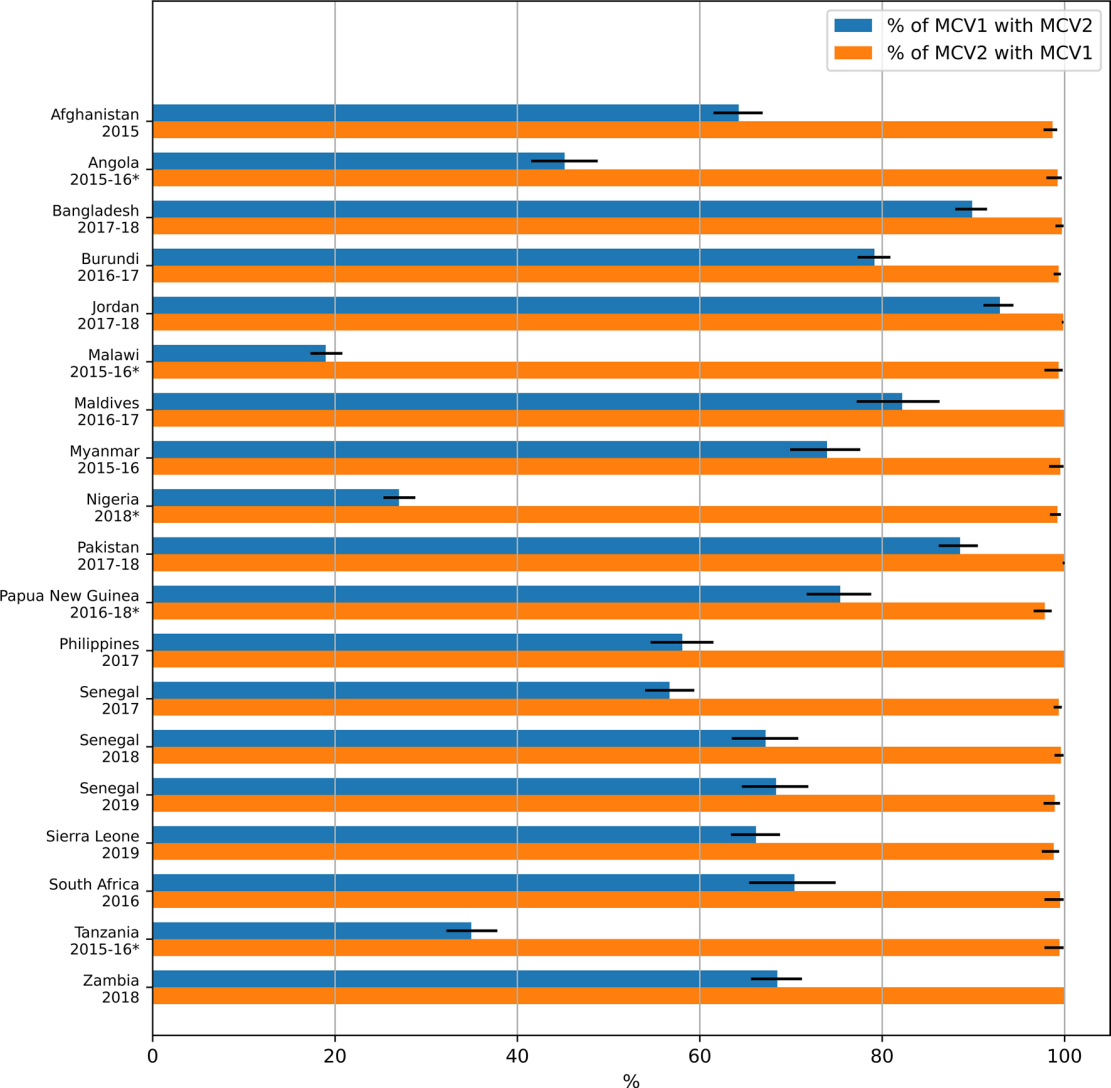
Percentage of children with probable MCV1 who also probably received MCV2 and of children with probable MCV2 who had also probably received MCV1. The numerical data depicted in the figure are also included in tabular form in the supplement (see Table S5). Surveys for which MCV2 was introduced less than 3 years before the survey are denoted with an asterisk.

Among the children with probable MCV1, the percentage of children with probable MCV2 was over 50% for all the countries which had introduced MCV2 at least 3 years before the survey, with the highest percentage (93%) estimated in Jordan (2017-18) (Figure 4 and Table S5, Supplement). It was, however, at 70% or less (i.e. dropout from MCV1 to MCV2 of 30% or more) in 8 surveys (6 countries) in which MCV2 was introduced at least 3 years before the survey. For the other countries, the percentage ranged from 19% (Malawi 2015-16) to 75% in Papua New Guinea (2016-18). Almost all of the children with probable MCV2 had probable MCV1 (Figure 4 and Table S5, Supplement). This percentage ranged from 98% in Papua New Guinea (2016-18) to 100% in several surveys. As is further discussed in the Discussion section, the definition of MCV2 and the structure of the DHS questionnaires should not allow for the data to include instances of children with MCV2 but not MCV1 and such occurrences should be attributed to errors in the data collection or data cleanup,

The ranges of these estimates were similar for most surveys when calculated using only data from children whose vaccination status was noted in the vaccination card (Figures S1 and S2, Table S6 in the Supplement). For 15 studies, the central estimate was higher when calculated using the vaccination card alone, compared to that based on either the vaccination card or mother’s report. For the remaining four surveys, (Burundi (2016-17), Malawi (2015-16), Nigeria (2018), and Tanzania (2015-16)), three of whom had introduced MCV2 during the three years before the surveys, the central estimate obtained using vaccination cards alone was lower than that estimated using both the vaccination card and the mother’s report.

In sensitivity analyses looking at regional differences, the percentage of probable MCV1 recipients who probably received MCV2 showed little regional variation in Pakistan (2017-18) (Figure S3 and Table S7 in the Supplement) but considerably more variation for Afghanistan (2015) ranging from 24% (95% CI: 5–64%) in Nooristan to 91% (95% CI: 82–96%) in Helmand (Figure S4 and Table S8 in the Supplement).

**Fig. 5:**
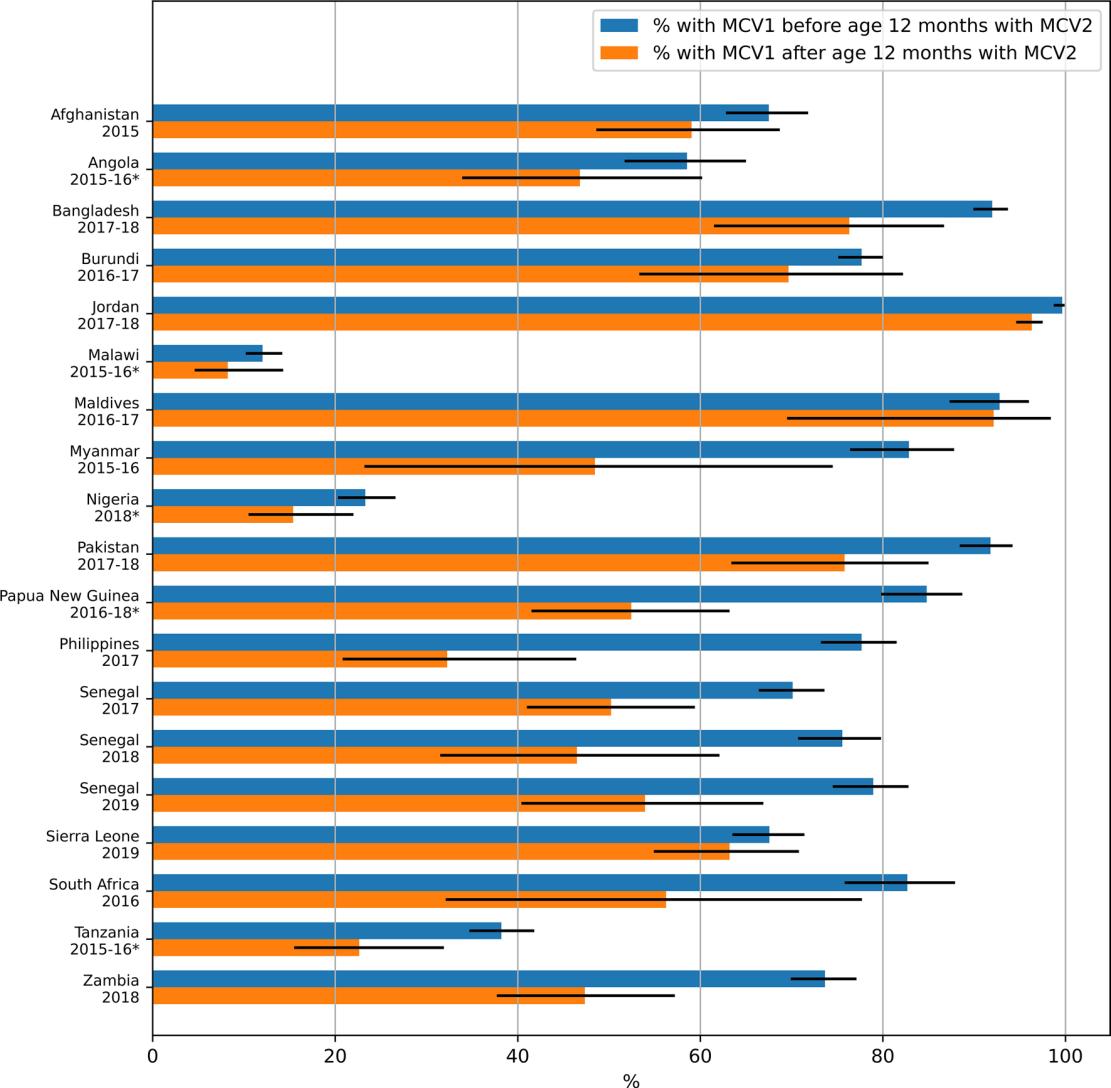
MCV1 status and percentage of children with probable MCV1 who also probably received MCV2 according to age at MCV1. The numerical data depicted in the figure are also included in tabular form in the supplement (see Table S9). Surveys for which MCV2 was introduced less than 3 years before the survey are denoted with an asterisk.

Figure 5 and Supplementary Table S9 show the association of age at MCV1 vaccination with the percentage of MCV1 recipients who went on to receive MCV2. The estimates were based on children whose vaccination status was determined from an existing and dated vaccination card and in all surveys, the percentage was higher for those who received MCV1 before age 12 months than for those who received it after age 12 months. The difference was statistically significant (*p* < 0.05) for 13 of the surveys (Supplement, Table S9), including some of the surveys for which MCV1 been introduced less than 3 years before the survey. In Pakistan (2017-18), of the children who had received MCV1 by age 12 months, 92% (95% CI: 88–94%) went on to receive MCV2, as compared with 76% (95% CI: 63–85%) of those who received MCV2 after age 12 months (*p* = 0.0025). Larger differences between the two percentages were seen elsewhere, e.g. 78% (95% CI: 73–81%) and 32% (95% CI: 21–46%), *p* < 0.0001, for those aged under and over 12 months respectively in Philippines (2017). In sensitivity analyses exploring regional differences, in Pakistan (2017-18), the country-wide trend was generally repeated in corresponding percentages for the ADM1 provinces but with wider confidence intervals due to the smaller sample sizes (Figure S5 and Table S10, Supplement). As a result, the difference between MCV1 recipients who also received MCV2 was statistically significant for different age of receipt of MCV1 at the *p* = 0.05 level for only two out of the six ADM1 provinces for which data was available. In contrast, in Afghanistan the association of age at MCV1 with the proportion of MCV1 recipients who subsequently received MCV2 varied with ADM1 province. The percentage of MCV1 recipients who then received MCV2 was higher for those who received MCV1 before age 12 months in 26 provinces and statistically significantly so in 10 of those provinces. That percentage was higher for children who received MCV1 after age 12 months in 5 provinces but that difference was not statistically significant in any of those cases. (Figure S6 and Table S11, Supplement).

**Fig. 6:**
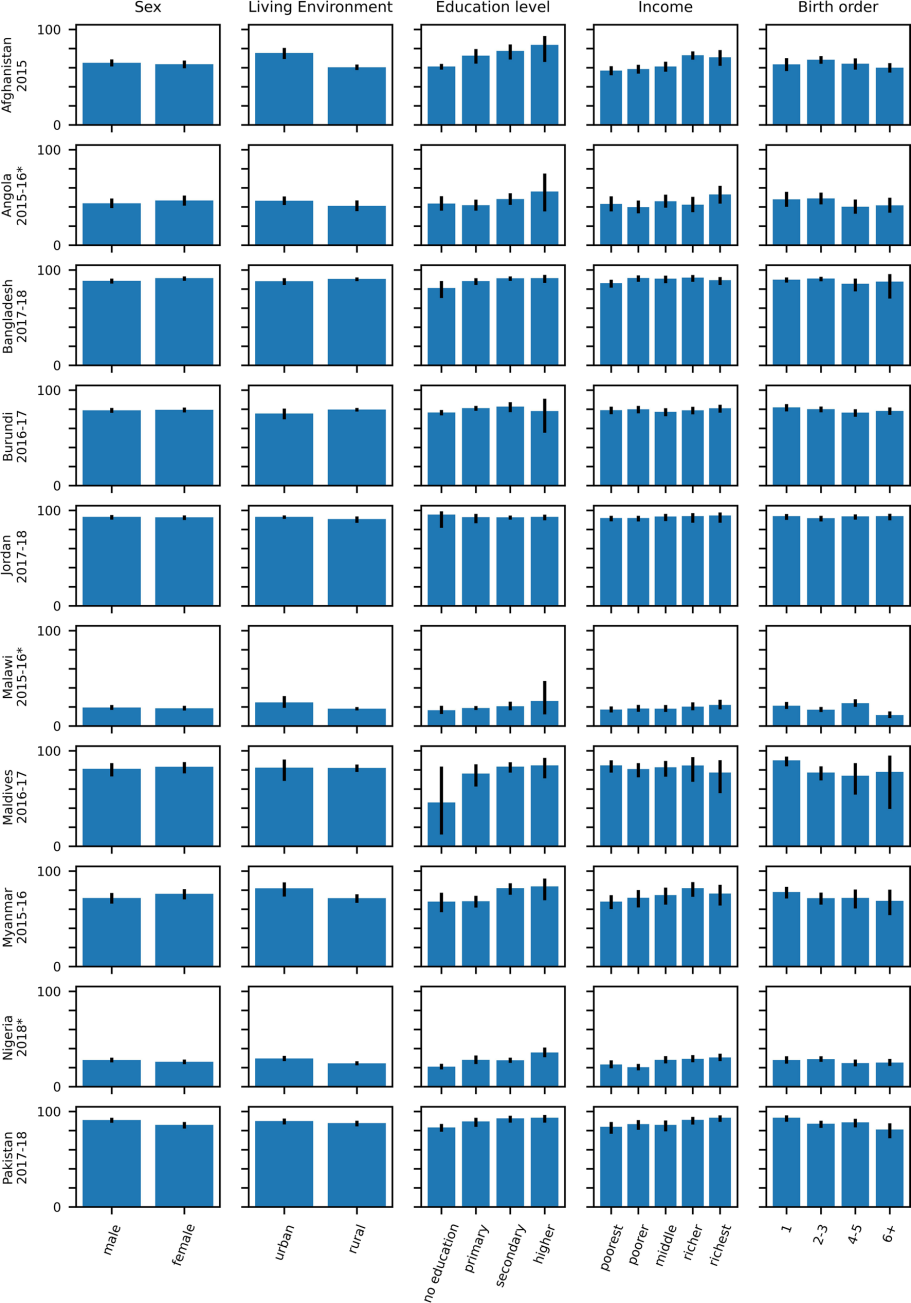
Percentage of children with probable MCV1 who probably went on to receive MCV2 stratified over the stratification categories described in the text with 95% CI (part 1–10 surveys). The numerical data depicted in the figure are also included in tabular form in the supplement (see Table S12). Surveys for which MCV2 was introduced less than 3 years before the survey are denoted with an asterisk.

**Fig. 7:**
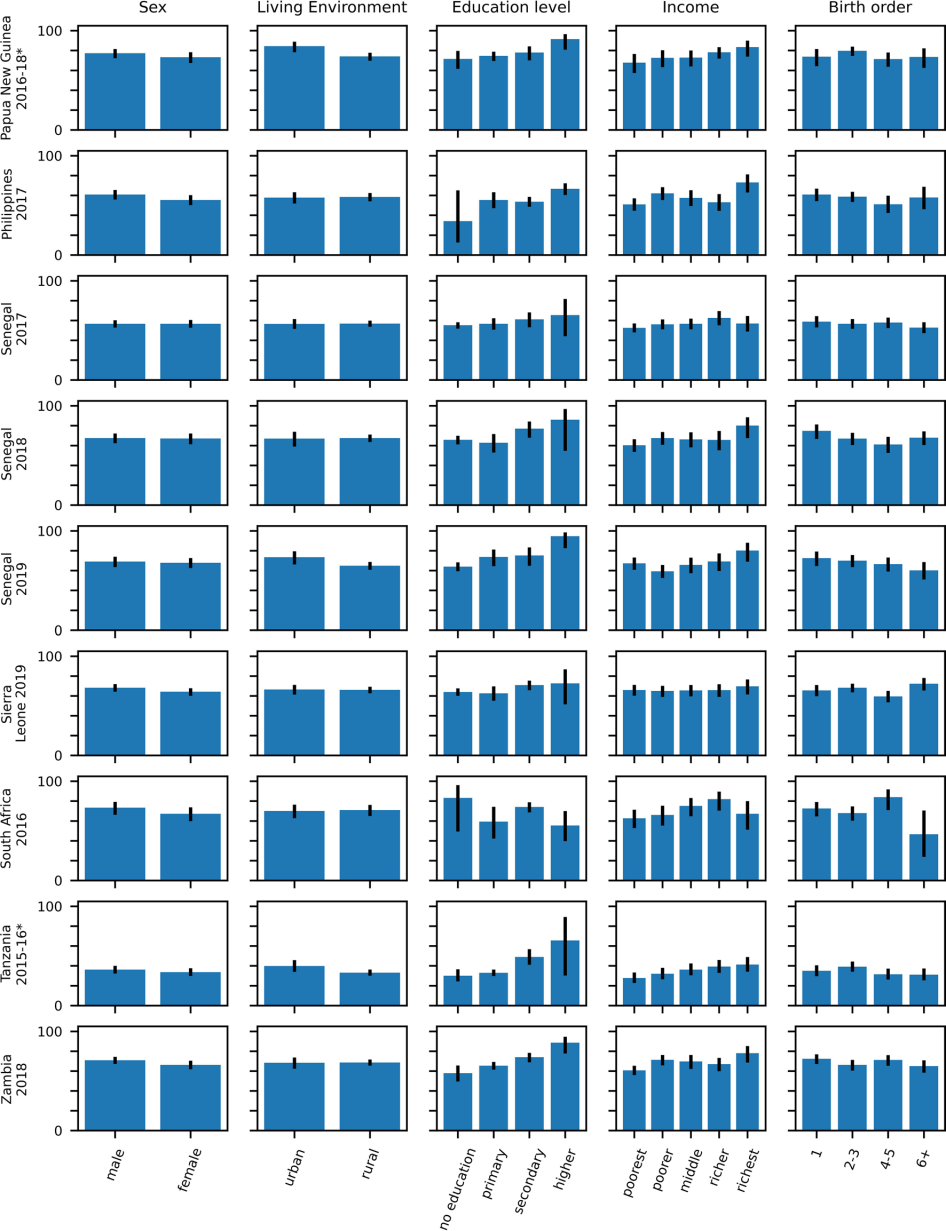
Percentage of children with probable MCV1 who probably went on to receive MCV2 stratified over the stratification categories described in the text with 95% CI (part 2–9 surveys). The numerical data depicted in the figure are also included in tabular form in the supplement (see Table S12). Surveys for which MCV2 was introduced less than 3 years before the survey are denoted with an asterisk.

Figures 6 and 7 and Table S12 (Supplement) present the percentage of children with probable MCV1 who then probably received MCV2 stratified over age, sex, urban and rural residency, income status and birth order. In general, for all surveys, the percentage was similar for each of the strata, except for education status and to a smaller degree income. In several surveys the percentage of children with probable MCV1 who then probably received MCV2 increased with increasing level of education of the mother and income level of the family. For example, for Zambia (2018), 58% (95% CI: 50–66%) of probable MCV1 recipients born to mothers with no education went on to receive probable MCV2, compared with 65% (95% CI: 61–69%), 74% (95% CI: 69–78%) and 89% (95% CI: 78–95%) of children born to mothers educated to primary, secondary and higher levels respectively.

**Fig. 8:**
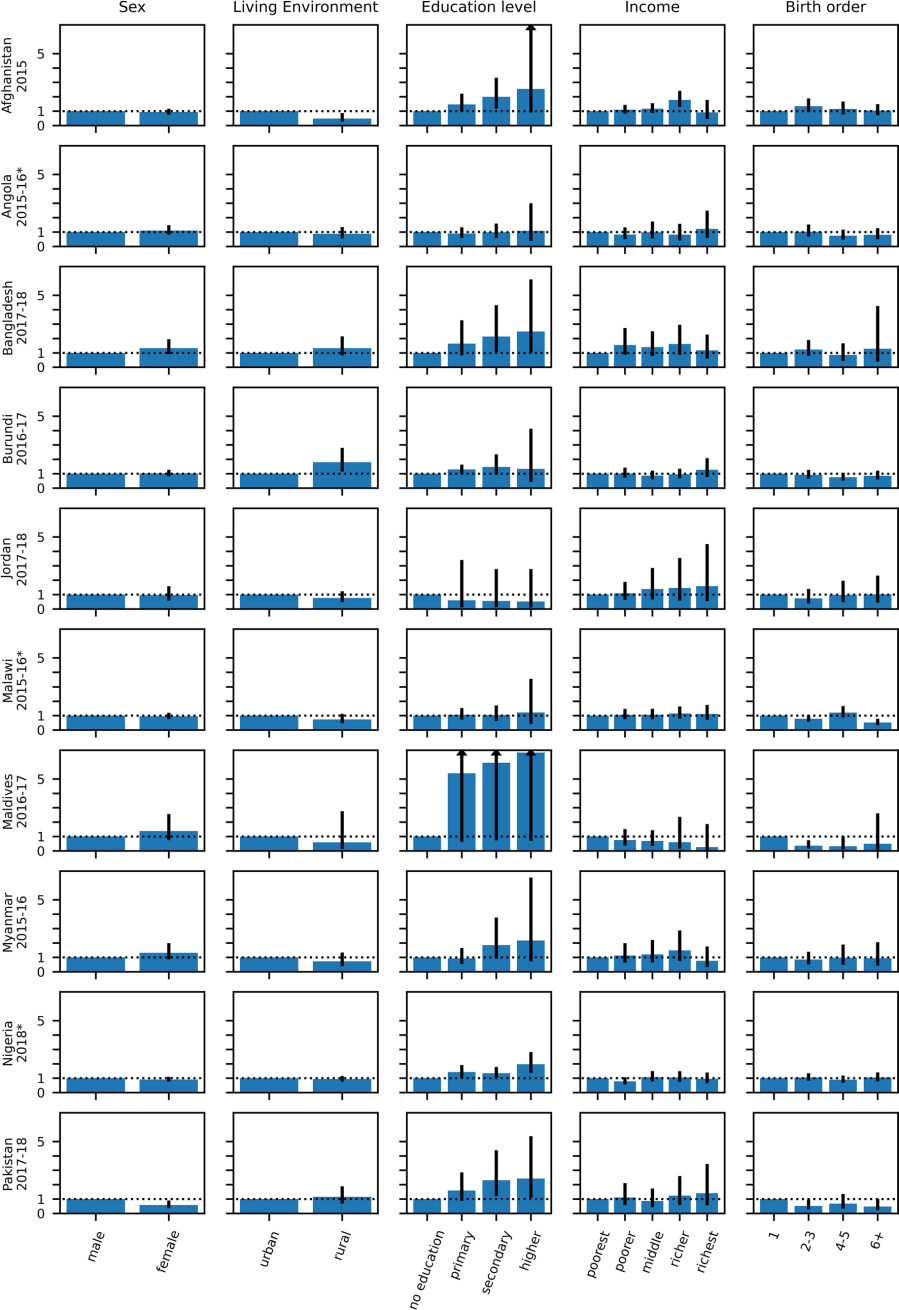
Adjusted Odds Ratios for the proportion of children with probable MCV1 who went on to probably receive MCV2 for the categories and strata described in the text with 95% CI (part 1–10 surveys). Arrows in the CI bars indicate that the higher CI range is outside of the plot range. The numerical data depicted in the figure are also included in tabular form in the supplement (see Table S12). Surveys for which MCV2 was introduced less than 3 years before the survey are denoted with an asterisk.

**Fig. 9:**
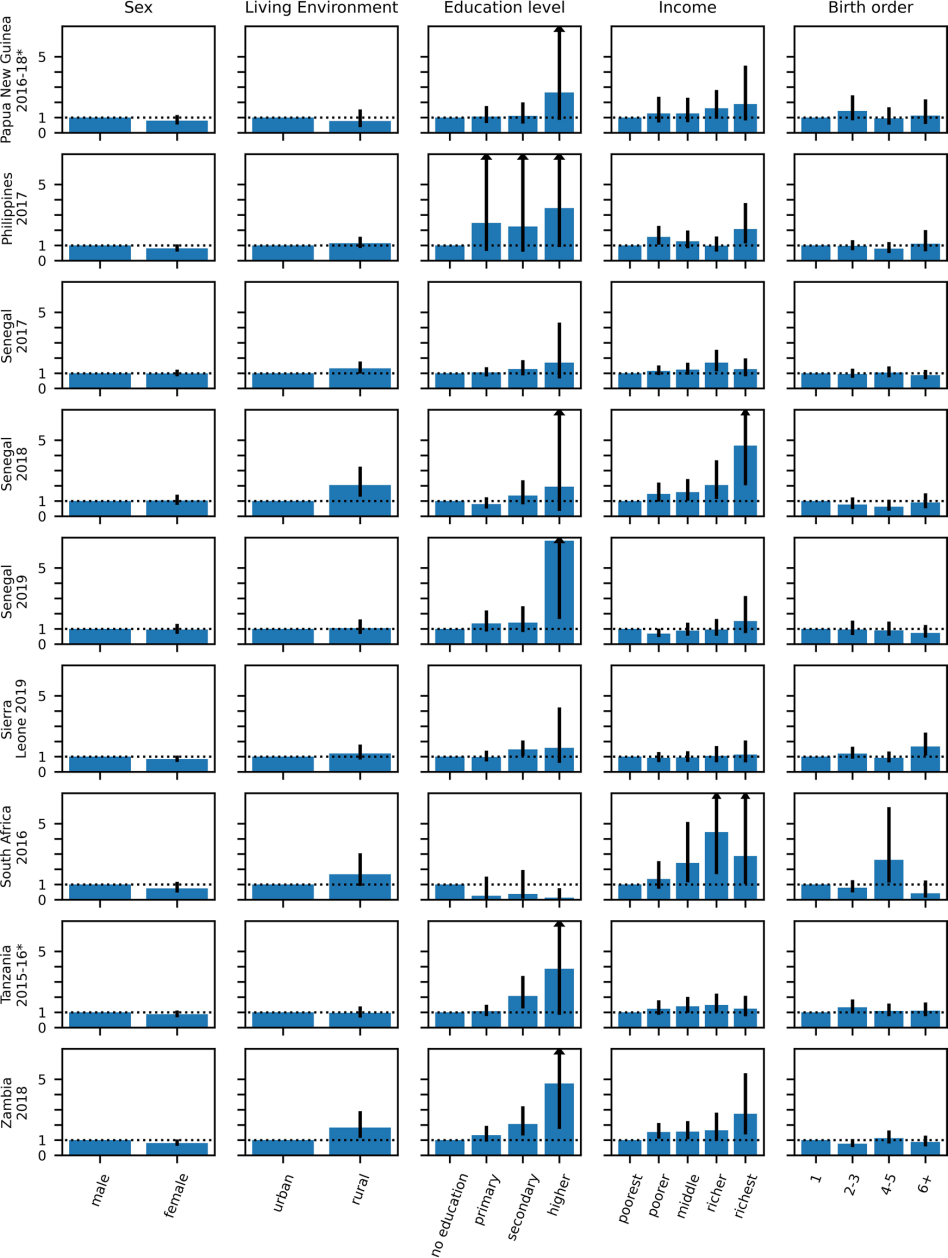
Adjusted Odds Ratios for the proportion of children with probable MCV1 who went on to probably receive MCV2 for the categories and strata described in the text with 95% CI (part 2–9 surveys). Arrows in the CI bars indicate that the higher CI range is outside of the plot range. The numerical data depicted in the figure are also included in tabular form in the supplement (see Table S12). Surveys for which MCV2 was introduced less than 3 years before the survey are denoted with an asterisk.

Figures 8 and 9 and Table S12 (Supplement) illustrate how different strata are associated with the percentage of probable MCV1 recipients who go on to probably received MCV2. In a multivariable regression model including the variables of age, sex, residency, mother’s education status and birth order, the mother’s education status was most likely to be associated with a statistically significant odds ratio for this percentage, with a higher percentage of probable MCV1 recipients born to mothers with some (primary, secondary or higher) education going on to probably receive MCV2 compared to those with mothers with no education. For example, considering children born to mothers with primary, secondary and higher education levels, the odds ratio compared to children born to mothers with no education was statistically significantly greater than 1 in 2, 7 and 5 surveys respectively (Tables 1 and 2 and Table S12). The odds ratio for children with mothers with higher education compared to those with mother with no education was less than 1 for just one survey - South Africa (2016). For Senegal (2019), the proportion of probable MCV1 recipients born to women in the higher education stratum who went on to probably receive MCV2 was over six times higher than that for children born to mothers with no education (aOR 6.8 (95% CI: 1.6–27.9)).

For the category of ‘Income’ the odds ratio was significantly greater than 1 in slightly fewer strata, namely in 2, 3, 4 and 4 surveys among children in families with income in the poorer, middle, richer and richest strata respectively. The highest adjusted odd ratio value was 4.64 (95% CI: 2.04–10.53) for the richest stratum in Senegal (2018).

The strata in the other categories were statistically significant considerably fewer times. The adjusted odds ratio for the association between birth order and the proportion of probable MCV1 recipients who also probably received MCV2 was inconsistent, being significantly less than unity in 2 and 1 surveys among children with birth order in the 2–3 and 6 + strata respectively, but significantly greater than 1 for one survey in the birth order 4–5 stratum and one in the birth order 6 + stratum (South Africa, (2016) and Sierra Leone (2016) respectively). The association of rural compared to urban residency stratification was also inconsistent, with the adjusted odds ratios for children living in rural areas being statistically significantly higher than unity in 3 surveys and lower than unity in 1 survey. Being female, compared to male was significantly associated with the proportion of probable MCV1 recipients who also probably received MCV2 only in one survey. Those results are summarised in Tables 1 and 2 and a more detailed listing including crude odds ratios and p-values over the same categories and stratification levels is included in tabular form in the supplement (Table S12).

**Table 1:**
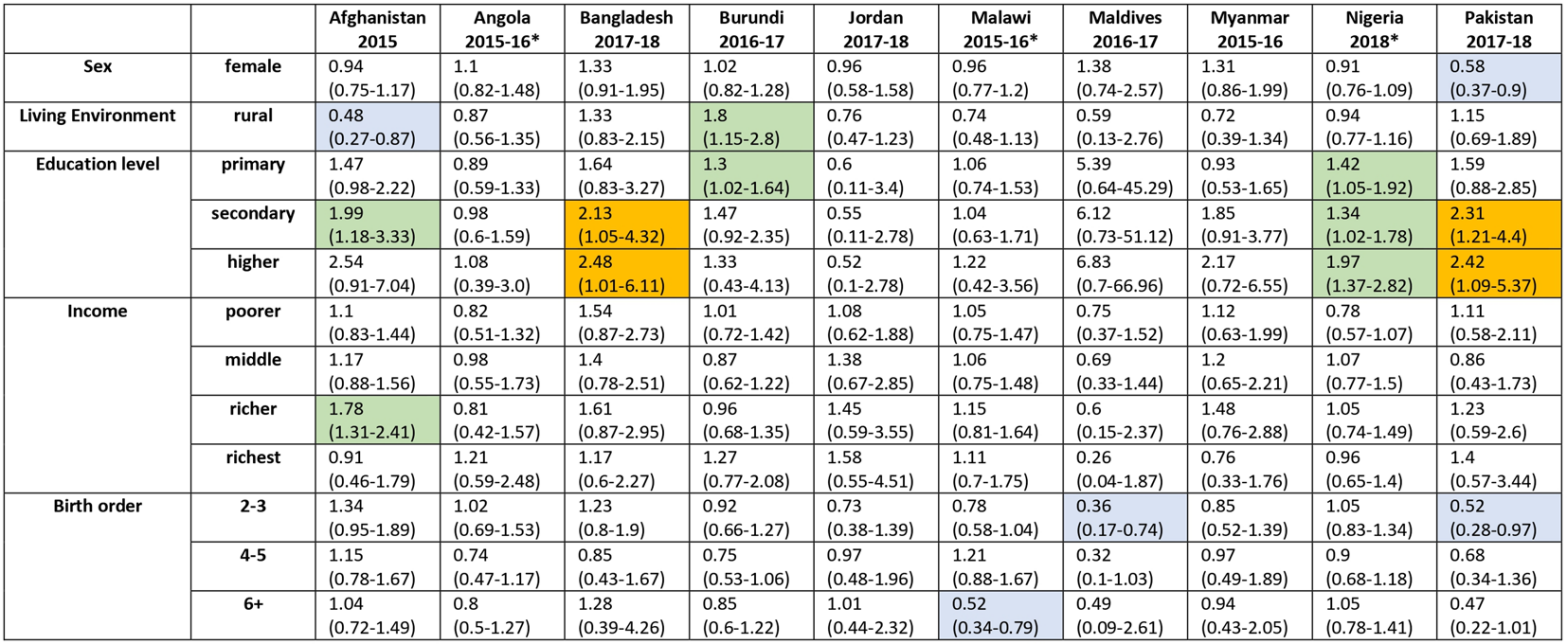
Summary of the adjusted odds ratio for the percentage of children with probable MCV1 who went on to probably receive MCV2 for all categories, compared to the corresponding baseline (part 1–10 surveys). The cells relating to surveys for which the adjusted OR was less than one and statistically different from 1 are shaded in blue; the cells relating to surveys for which the adjusted OR was between 1 and 2 and statistically significantly different from 1 are in green; the cells relating to surveys for which the adjusted OR was > 2 and statistically significantly different from 1 are in orange. Surveys for which MCV2 was introduced less than 3 years before the survey are denoted with an asterisk.

**Table 2:**
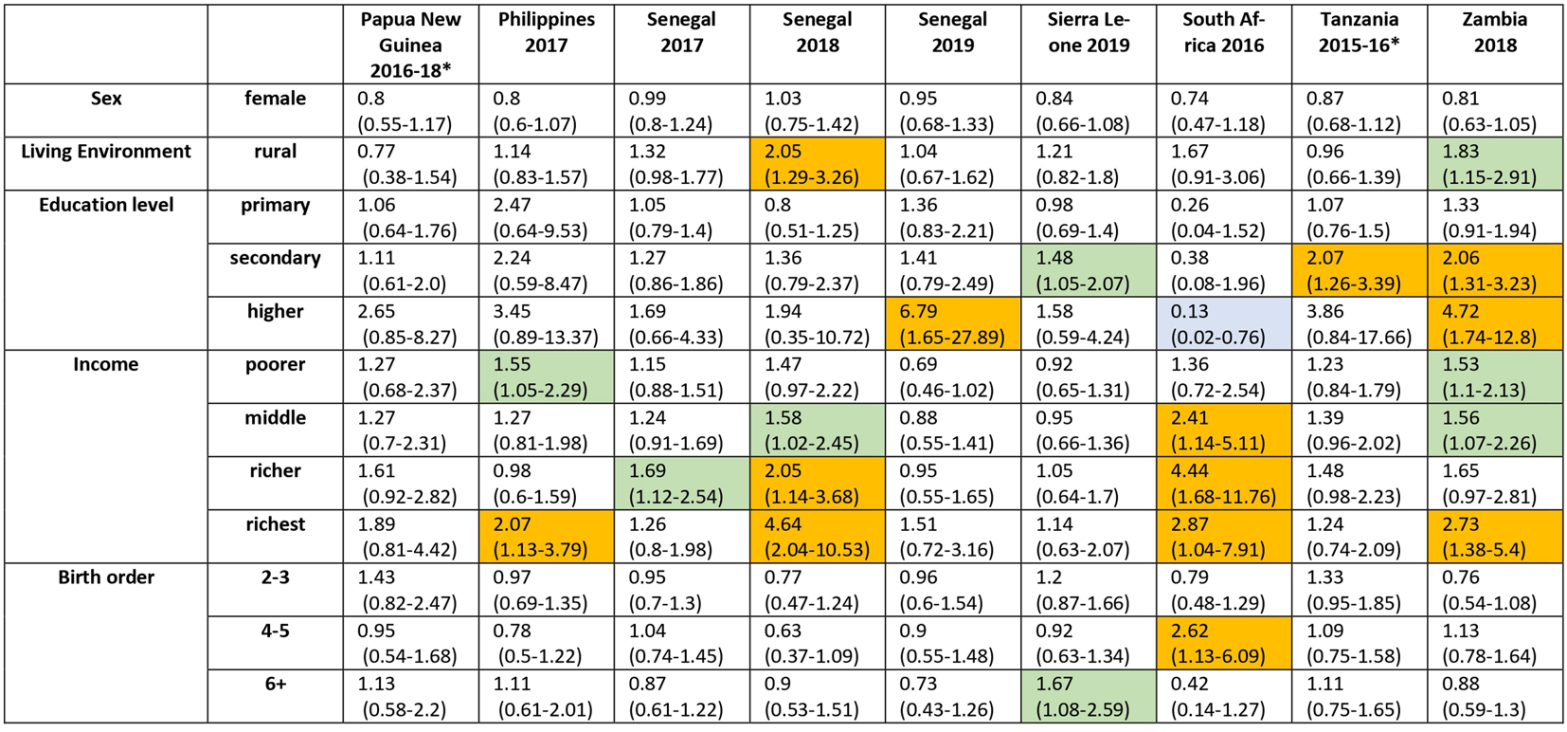
Summary of the adjusted odds ratio for the percentage of children with probable MCV1 who went on to probably receive MCV2 for all categories compared to the corresponding baseline (part 2–9 surveys). The cells relating to surveys for which the adjusted OR was less than one and statistically different from 1 are shaded in blue; the cells relating to surveys for which the adjusted OR was between 1 and 2 and statistically significantly different from 1 are in green; the cells relating to surveys for which the adjusted OR was > 2 and statistically significantly different from 1 are in orange. Surveys for which MCV2 was introduced less than 3 years before the survey are denoted with an asterisk.

## Discussion

We used DHS data to calculate the percentage of probable MCV1 recipients who probably received MCV2 and factors that are associated with this percentage in 19 surveys. We found that over 50% of probable MCV1 recipients were likely to have also received MCV2 in all of the surveys in which MCV2 had been in place for at least three years. This percentage was very high (over 88%) in 3 surveys, namely Bangladesh (2017-18), Jordan (2017-18) and Pakistan (2017-18). For several surveys, the percentage increased significantly with the level of education attained by the mother and the income of the household. However, the dropout rate from MCV1 to MCV2 is still very high for most of the surveys examined here, highlighting the need to strengthen vaccination in the second year of life and beyond^29,30^.

We found that, in many settings, the percentage of children with probable MCV1 who then probably received MCV2 was positively associated with the level of education attained by the mother or the income status and that in some settings, the level of education attained by the mother was more strongly associated with this percentage than the income status. Our findings highlight population groups that, if targeted, are more likely to improve the proportion of children that have been fully-vaccinated for measles. Our findings are also consistent with those from other surveys which considered vaccination coverage and/or other vaccines. For example, one recent literature review^31^ found that children of mothers with no education or those in the lowest income group were less likely to be fully-vaccinated for several vaccines than those with primary or higher education and higher income groups respectively. Other studies which considered two regions in Ethiopia have also found that the increasing level of education of the mother was the most strongly associated determinant for reduced dropout from MCV1 to MCV2^18^ and from Pentavalent vaccination to MCV1^17^. In a study considering 8 countries in sub-Saharan Africa^32^ the level of education of the mother was the determinant most strongly associated with MCV2 uptake. Those studies included a set of determinants with more sociodemographic characteristics than the ones we considered in this study as well as additional health service utilization and vaccination information characteristics.

A particularly strong association between the mother’s education and the proportion of probable MCV1 recipients who went on to probably receive MCV2 was found in Nigeria, where at the time of the DHS survey, MCV2 had yet to be added to the routine schedule. The children who had received MCV2 are likely to have received it in the private sector, which is probably more likely to be accessed by mothers with a higher educational level than those with no education.

Most of the analyses we presented considered MCV1 and MCV2 at the country level, thus masking any heterogeneity within the survey settings. However, we conducted analysis at the regional level for two surveys, Afghanistan (2015) and Pakistan (2017-18). In Afghanistan, the percentage of children with probable MCV1 who went on to probably receive MCV2 and the relation of that percentage with the age of receiving MCV1 varies considerably across different ADM1 provinces and the difference was statistically significant for several provinces. The same analysis for Pakistan (2017-18) showed much less geographic variation. Such analysis was not meaningful across all 19 datasets due to limited size of most of the datasets. Using spatial data from DHS for five countries, another study^33^ found differences both between and within countries for measles and diphtheria-pertussis-tetanus (DPT) vaccination coverage and in the drop-out in the uptake of subsequent doses for DPT following the first dose. Uptake was associated with various factors, depending on the country, such as distance or travel time to urban areas. In a more recent study^24^ studying 9 countries, Utazi et al. found that the education level of the mother is one of the strongest predictors for MCV1 uptake. As such, improving coverage in particular population groups may require both increasing their awareness of the benefits of receiving subsequent doses of vaccine and potentially improving access to the health centres providing the vaccine.

In our analyses, we defined children with ‘probable’ vaccination status as those who were reported to have been vaccinated either on the vaccination card or by the mother. This simplified definition is unlikely to have affected many of our findings, given that our estimates of the percentage of children with probable MCV1 who went on to probably receive MCV2, remained relatively unchanged even when the data from children whose vaccination had been only reported by the mother were excluded from the analysis (Figure S2 and Table S6). However, we have not investigated the degree to which the association of this percentage with age, sex, residency, mother’s education, wealth and birth order may be affected by excluding the cases where vaccination is only reported by the mother. Our estimates of this association could have been affected by excluding records where vaccination is only reported by the mother, given that recent research suggests that the ownership of vaccination cards in households in low-income and middle-income countries is associated with household wealth and maternal schooling^34^.

Using data from children whose vaccination status was determined from vaccination cards alone (Figure S1), we found that almost all probable recipients of MCV2 had previously probably received MCV1. This indicates that (consistent with the WHO definitions of MCV2 coverage^35^) vaccine providers record a vaccine dose on the vaccination card as MCV2 only if the recipient is receiving the second dose, and the first vaccine dose is recorded as being MCV1 even if it was provided at the age at which MCV2 would have been provided. As the DHS questionnaire^36^ is structured, it appears to allow the recording of MCV2 without MCV1 if a vaccination card with such information is seen by the interviewer (Question 509 of the questionnaire). For children without vaccination cards, designation of their MCV1 and MCV2 status in DHS surveys is determined from the mother’s answer to Question 528 “How many times did (NAME IN 503) receive the measles vaccine?”. For a small number of records (13 across all the surveys, data not shown), children were reported as having received MCV2 without MCV1 based on the report of the mother. These records could have resulted from errors made whilst recording by the vaccinator or during transcription in the questionnaire or compilation of the DHS data files.

We found that, compared to those who had received MCV1 before age 12 months, a smaller proportion of those aged over 12 months when receiving MCV1 went on to receive MCV2, a finding that was statistically significant for 13 out of the 19 surveys for which this information was available (see Table S9). Whilst it is plausible that age at vaccination influences whether or not children receive both vaccine doses, this finding could be partly attributable to other factors. For example, these estimates were based just on children whose date of vaccination was recorded on a vaccination card, and it is unclear whether they are representative of children whose date of vaccination is unknown. In addition, our analyses were restricted to children who were aged 24–35 months at the time of the survey. Consequently, any children who received MCV1 at the upper end of this age range and received MCV2 after age 35 months would have been recorded as only having received MCV1 in our analysis. However, this may not have affected our estimates substantially, given the small number of children who received MCV1 after age 24 months (Figure 3), at least among those whose date was recorded on their vaccination card. It is also possible that children did not return for MCV2 vaccination if they had missed the opportunity for MCV1 and came to be vaccinated at the time scheduled for MCV2. Such children are likely to have been reported as having received MCV1, as they should be.

Our analyses were subject to several limitations. Out of the 315 DHS surveys, only 19 were found to have collected data on MCV1 and MCV2 and were used in our analyses to consider the overlap between MCV1 and MCV2 recipients. It is unclear if the countries represented in these 19 surveys are representative of other countries. Furthermore, we included five countries in which MCV2 was introduced less than 3 years before the corresponding survey, including one survey in a country which had not yet introduced MCV2, so the children reported to have received MCV2 could have been vaccinated in the private sector. This is consistent with our finding that the distribution of the age at MCV2 vaccination for these countries was inconsistent with the scheduled age (see Figure 3 and relevant analysis in the ‘Results’ section), given that the age at vaccination in the private sector is likely to be more flexible than in the public sector. Except for Papua New Guinea (2016-18), the percentages of children with probable MCV1 who went on to probably receive MCV2 were the lowest in those surveys. Our estimates could have been affected by recall error by the mother given our definition of probable MCV1 and probable MCV2 recipients. Such recall errors would have affected some countries more than others. For example, 16% of the probable MCV1 recipients were defined based just on the mother’s report in Maldives (2016-17), as compared with 63% in Myanmar (2015-16) and Nigeria (2018) (Supplement, Table S2). Our analysis was restricted to the data available before the COVID pandemic and it is plausible that some values may be changed following the COVID pandemic. Also, other sources of similar data are currently becoming available (e.g. data from the UNICEF’s Multiple Indicator Cluster Surveys programme^37^) which can be used in future work to provide more detailed answers to the questions posed here. Our analyses did not separately quantify the impact of vaccination received through Supplementary Immunization Activities (SIAs). In instances where the vaccination card is not present or it does not include information about both doses of measles vaccination, information based on the mother’s recall is recorded without differentiating if vaccination was given as part of a campaign. Some DHS surveys include information related to vaccination received through campaigns in a “vaccinated during campaign” indicator (see for example the analysis in^14^) but we have not included any such information in our analyses. Finally, our analyses did not take into account the possible effect of active conflict at the time of the survey in some of the countries that we have included in our analyses. It should be expected that active conflict should have a significant effect on the representation of clusters and Primary Sampling Units (PSUs) included in the DHS data and potentially in the opportunity for children to receive vaccination according to the national schedule.

Maintaining and improving vaccination coverage is essential for the control and potential elimination of measles and rubella across the world. One of the strategic priorities of IA2030 is that immunization is valued and actively sought by all people. Our analyses have highlighted important differentials in shortfalls by age, country, mother’s education and income status in the proportion of recipients of MCV1 who go on to receive MCV2. Targeting those differentials is essential if the goals of measles elimination are to be achieved.

## Methods

### Data sources

We used data from the Demographic and Health Surveys (DHS) Program (https://dhsprogram.com/). The DHS provides a wide range of data on population, health and economic indicators from household interview surveys conducted in over 90 countries in different years during the period 1985–2019. These include measles vaccination coverage as determined by the number of biological children of women of reproductive age in the visited households that have received either one or two doses of measles vaccination at any time before the survey, as well as the date of the vaccination administration. According to the DHS interviews protocol, this information for each household was obtained from the children’s vaccination card together with the date of the vaccination administration if that information was present in the card. When that information was not available from the vaccination card, the answer obtained by questioning the mother of the child was recorded.

### Statistical analyses

Our analyses were restricted to data from children who were aged 24–35 months at the time of the DHS survey, and to the surveys which had collected data on receipt of the first and second doses of measles vaccination of survey participants (coded as h9 and h9a respectively in the DHS survey). After examining all 315 surveys available from DHS (data downloaded in April 2021), we identified 19 surveys that have data meeting these specifications (Supplement, Table S1). For all of the countries included in those surveys, MCV1 was scheduled during the first year of life of the child and MCV2 was scheduled during the second year of life of the child (Supplement, Table S4^25^). Restricting our analysis to children aged 24–35 months in these surveys results in inclusion of only children who have had the opportunity to receive both doses of measles vaccination if MCV2 vaccination is already in place at the time of the survey. Three of the countries included in our analysis have additional scheduled dose(s) of measles containing vaccination (Jordan at 18 months, Papua New Guinea at 7 years and Philippines at 5–6 years and 12–13 years, see Table S4 in the supplement and reference^25^). For 14 of the surveys included in our analyses, the year of introduction of MCV2^38^ was at least 3 years before the year of the survey. In the remaining 5 surveys MCV2 was introduced in the year before the survey started for one survey (Tanzania (2015-16), in the same year as the survey started for three of the surveys (Angola (2015-16), Malawi (2015-16) and Papua New Guinea (2016-18)) and about 2 years after the start of the survey for one survey (Nigeria (2016). Further details are provided in Table S13. We included those 5 surveys for which MCV2 had been introduced less than 3 years before the survey as there was a sizeable proportion of children for which MCV2 vaccination information could be extracted from the DHS data.

In the DHS data, the variable relating to receipt of MCV1 is coded as either ‘No’, ‘Yes, date on vaccination card’, ‘Yes, from mother’s report’, ‘Yes, from vac card w/out date’, ‘Don’t know’. The categorisations relating to receipt of MCV2 were similar. For the purposes of our analyses, we defined children as having “probably received MCV1” (or equivalently as “probable MCV1” recipients) if their MCV1 vaccination status was coded as either ‘Yes, date on vaccination card’, or ‘Yes, from mother’s report’, or ‘Yes, from vac card w/out date’. We defined children who have “probably received MCV2” (equivalently “probable MCV2” recipients) similarly. We also extracted the information about the date of MCV1 vaccination and date of birth of the child (coded as h9d, h9m, h9y and b18 in the DHS survey).

For each of the 19 surveys we computed the following proportions:

- The proportion of children with probable MCV1 who subsequently also probably received MCV2.
- The proportion of children with probable MCV2 who had previously also probably received MCV1. Calculation of this proportion helps with identifying the extent to children reported to be recipients of MCV2 have received two measles vaccine doses.
- The proportion of children who received MCV1 by age 12 months (based on vaccination card) who then probably received MCV2.
- The proportion of children who received MCV1 after age 12 months (based on the vaccination card) who then probably received MCV2.
- The proportions of children who received (a) only probable MCV1 and not MCV2, (b) only probable MCV2 but not MCV1 and (c) neither MCV1 nor MCV2.

In sensitivity analyses of the effect of our definition of “probable recipients of MCV”, we recomputed the first two proportions just using records from those children who had their vaccination reported on their vaccination card (either with or without vaccination date information). For each of the above proportions we also computed the 95% confidence intervals. We also extracted the WUENIC coverage estimates^39,40^ for the year in which the children covered by the survey were 1 year old (for MCV1) or 2 years old (for MCV2), i.e. respectively 2 and 1 years before the year or years of the survey.

For two of the most populous countries in our dataset, Afghanistan and Pakistan, we computed the first four of the above listed proportions by region of residence of the household, using the first level administrative boundaries (ADM1 level, v024 variable in the DHS dataset).

The proportion of children with probable MCV1 who subsequently also probably received MCV2 vaccination was further stratified by sex, residency (urban or rural), educational status of the mother (defined as no education, primary, secondary, higher), wealth quintile of the household (defined as poorest, poorer, middle, richer and richest), and birth order of the child (defined as 1, 2–3, 4–5 and 6+) by use of the information coded in the ‘b4’, ‘v025’, ‘v106’, ‘v190’ and ‘bord’ variables in the DHS surveys. We computed the Adjusted Odds Ratios (adjusted for age, sex, residency, mother’s educational status wealth quintile and child’s birth order) and p-values for those strata using multivariable logistic regression.

The data analysis and presentation methodology was informed by a recently proposed standard for the methodology of reporting analyses of survey data (PRICSSA^41^). It follows the main points of that proposed standard (information about data collection dates and modes, target population, sample design, confidence intervals, weighting and variance estimation) but it excludes some other points (survey response rates, missingness rates and presentation of unweighted data).

### Data processing and software

We used modified versions of the Stata scripts that are provided by DHS (publicly available from https://github.com/DHSProgram/DHS-Indicators-Stata), executing those scripts from within a Python Jupyter Notebook environment, which invoked Stata version 16. We used Stata’s ‘svy’ command with the ‘subpop’ option to restrict our analysis to living children of the appropriate age group and vaccination and demographic characteristics for each part of the analysis. This method applies the appropriate weighting to the relevant tabulations according to the DHS stratified sampling design^42^. We obtained 95% confidence intervals using Stata’s implementation of the Taylor linearized variance estimation (‘vce linearized’). We used the *‘logistic’* command of Stata to fit a multivariable logistic regression model to the stratified data of the proportion of children with probable MCV1 who went on to receive probable MCV2 for each survey. In this way we calculated adjusted odds ratios (together with 95% confidence intervals and p-values) of the impact to that proportion of each of the strata in the categories sex, residency, education, income and birth order compared to the reference strata ‘male’, ‘urban’, ‘no education’, ‘poorest’, and ‘birth order’ respectively. Unless otherwise stated, statistical significance in our results is tested at the level of *p* = 0.05.

## Electronic supplementary material

Below is the link to the electronic supplementary material.

Supplementary Material 1

## Data Availability

The data that support the findings of this study are available from the Demographic and Health Surveys (DHS) Program but restrictions apply to the availability of these data, which were used under license for the current study, and so are not publicly available. Data are however available from the corresponding author upon reasonable request and with permission of the Demographic and Health Surveys (DHS) Program.

## References

[1] World Health Organization = Organisation mondiale de la. Measles vaccines: WHO position paper – April 2017. *Wkly. Epidemiol. Rec*. **92**, 205–227 (2017).28459148

[2] WHO. *Table 2: Summary of WHO Position Papers - Recommended Routine Immunizations for Children*, <https://www.who.int/publications/m/item/table-2-summary-of-who-position-papers-recommended-routine-immunizations-for-children

[3] World Health, O. *Measles and rubella strategic framework: 2021–2030*. x, 35 pWorld Health Organization,. (2020).

[4] WHO. *Immunization Agenda 2030: A Global Strategy to Leave No One Behind*, <https://www.who.int/teams/immunization-vaccines-and-biologicals/strategies/ia2030 10.1016/j.vaccine.2022.11.042 39004466

[5] Ou, A. C. et al. Progress toward rubella and congenital rubella syndrome elimination - Worldwide, 2012–2022. *MMWR Morb. Mortal. Wkly. Rep.* **73**, 162–167. 10.15585/mmwr.mm7308a2 (2024). 38421933 10.15585/mmwr.mm7308a2 PMC10907039

[6] Minta, A. A. et al. Progress toward measles elimination - Worldwide, 2000–2023. *MMWR Morb. Mortal. Wkly. Rep.* **73**, 1036–1042. 10.15585/mmwr.mm7345a4 (2024). 39541251 10.15585/mmwr.mm7345a4 PMC11576049

[7] WHO. *Immunization Agenda 2030 Scorecard*, <https://scorecard.immunizationagenda2030.org/

[8] UNICEF. Briefing note #6 Universal health coverage GOAL 3 Ensure healthy lives and promote well-being for all at all ages. (2018).

[9] Mckee, A., Ferrari, M. J. & Shea, K. Correlation between measles vaccine doses: Implications for the maintenance of elimination. *Epidemiol. Infect.* **146**, 468–475. 10.1017/S0950268817003077 (2018). 29465027 10.1017/S0950268817003077 PMC5848754

[10] Eilertson, K. E., Fricks, J. & Ferrari, M. J. Estimation and prediction for a mechanistic model of measles transmission using particle filtering and maximum likelihood estimation. *Stat. Med.* **38**, 4146–4158. 10.1002/sim.8290 (2019). 31290184 10.1002/sim.8290 PMC6771900

[11] Mc, K. A., Shea, K. & Ferrari, M. J. Optimal vaccine schedules to maintain measles elimination with a two-dose routine policy. *Epidemiol. Infect.* **145**, 227–235. 10.1017/S0950268816002296 (2017). 27760574 10.1017/S0950268816002296 PMC5197928

[12] Winter, A. K. et al. Feasibility of measles and Rubella vaccination programmes for disease elimination: a modelling study. *Lancet Glob Health*. **10**, e1412–e1422. 10.1016/S2214-109X(22)00335-7 (2022). 36113527 10.1016/S2214-109X(22)00335-7 PMC9557212

[13] World Health Organization, Organisation mondiale de la. Rubella vaccines: WHO position paper – July 2020 – Note de synthèse: position de l’oms concernant les vaccins Antirubéoleux. *Wkly. Epidemiol. Rec.* **95**, 306–324 (2020).

[14] Portnoy, A., Jit, M., Helleringer, S. & Verguet, S. Impact of measles supplementary immunization activities on reaching children missed by routine programs. *Vaccine* **36**, 170–178. 10.1016/j.vaccine.2017.10.080 (2018). 29174680 10.1016/j.vaccine.2017.10.080 PMC5949217

[15] Dansereau, E., Brown, D., Stashko, L. & Danovaro-Holliday, M. C. A systematic review of the agreement of recall, home-based records, facility records, BCG scar, and serology for ascertaining vaccination status in low and middle-income countries. *Gates Open. Res.* **3**, 923. 10.12688/gatesopenres.12916.2 (2019). 32270134 10.12688/gatesopenres.12916.1 PMC7110941

[16] Masresha, B. G. et al. Introduction of the second dose of measles containing vaccine in the childhood vaccination programs within the WHO Africa Region - Lessons learnt. *J Immunol. Sci Suppl.*, 113–121 (2018). PMC6372060 30766972

[17] Hailu, C., Fisseha, G. & Gebreyesus, A. Determinants of measles vaccination dropout among 12–23 months aged children in pastoralist community of Afar, Ethiopia. *BMC Infect. Dis.* **22**, 376. 10.1186/s12879-022-07350-1 (2022). 35421952 10.1186/s12879-022-07350-1 PMC9008940

[18] Adugna, B., Tola, A., Fite, M. B. & Motuma, A. Determinants of second-dose measles vaccination dropout in Ethiopia: A community-based matched case-control study. *Heliyon* **10**, e30764. 10.1016/j.heliyon.2024.e30764 (2024). 38756559 10.1016/j.heliyon.2024.e30764 PMC11096893

[19] Cockcroft, A. et al. One size does not fit all: Local determinants of measles vaccination in four districts of Pakistan. *BMC Int. Health Hum. Rights* (Suppl 1), S4–S4. 10.1186/1472-698X-9-S1-S4 (2009). 19828062 10.1186/1472-698X-9-S1-S4 PMC3226236

[20] Metcalf, C. J. E. et al. Transport networks and inequities in vaccination: Remoteness shapes measles vaccine coverage and prospects for elimination across Africa. *Epidemiol. Infect.* **143**, 1457–1466. 10.1017/S0950268814001988 (2015). 25119237 10.1017/S0950268814001988 PMC4411642

[21] Smith, P. J., Chu, S. Y. & Barker, L. E. Children who have received no vaccines: Who are they and where do they live?. *Pediatrics* **114**, 187–195. 10.1542/peds.114.1.187 (2004). 15231927 10.1542/peds.114.1.187

[22] Toikilik, S. et al. Are hard-to-reach populations being reached with immunization services? Findings from the 2005 Papua New Guinea National immunization coverage survey. *Vaccine* **28**, 4673–4679. 10.1016/j.vaccine.2010.04.063 (2010). 20451641 10.1016/j.vaccine.2010.04.063

[23] Wigley, A. et al. Estimates of the number and distribution of zero-dose and under-immunised children across remote-rural, urban, and conflict-affected settings in low and middle-income countries. *PLoS Glob. Public Health* **2**, e0001126. 10.1371/journal.pgph.0001126 (2022). 36962682 10.1371/journal.pgph.0001126 PMC10021885

[24] Utazi, C. E. et al. Assessing the characteristics of un- and under-vaccinated children in low- and middle-income countries: A multi-level cross-sectional study. *PLoS Glob. Public. Health* **2**, e0000244. 10.1371/journal.pgph.0000244 (2022). 36962232 10.1371/journal.pgph.0000244 PMC10021434

[25] WHO. *Vaccination schedule for Measles*, https://immunizationdata.who.int/pages/schedule-by-disease/measles.html?ISO_3_CODE=AFG+AGO+BGD+BDI+JOR+MMR+MDV+MWI+NGA+PNG+PHL+PAK+SLE+SEN+TZA+ZAF+ZMB&TARGETPOP_GENERAL=

[26] WHO. *WHO/UNICEF Joint Reporting Process*, https://www.who.int/teams/immunization-vaccines-and-biologicals/immunization-analysis-and-insights/global-monitoring/who-unicef-joint-reporting-process

[27] DHS. Papua New Guinea Demographic and Health Survey 2016-18. National Statistical Office Port Moresby, Papua New Guinea, Rockville, Maryland, USA, (2019).

[28] DHS. *South Africa Demographic and Health Survey 2016* (National Department of Health, 2019).

[29] WHO. *Vaccination in the second year of life (2YL)*, https://www.who.int/teams/immunization-vaccines-and-biologicals/essential-programme-on-immunization/integration/vaccination-in-the-second-year-of-life-(2yl)

[30] O’Brien, K. L. & Lemango, E. The big catch-up in immunisation coverage after the COVID-19 pandemic: progress and challenges to achieving equitable recovery. *Lancet* **402**, 510–512. 10.1016/S0140-6736(23)01468-X (2023). 37478887 10.1016/S0140-6736(23)01468-X

[31] Ali, H. A. et al. Vaccine equity in low and middle income countries: A systematic review and meta-analysis. *Int. J. Equity Health* **21**, 82. 10.1186/s12939-022-01678-5 (2022). 35701823 10.1186/s12939-022-01678-5 PMC9194352

[32] Chilot, D. et al. Measles second dose vaccine utilization and associated factors among children aged 24–35 months in sub-Saharan Africa, a multi-level analysis from recent DHS surveys. *BMC Public Health* **22**, 2070. 10.1186/s12889-022-14478-x (2022). 36371164 10.1186/s12889-022-14478-x PMC9655865

[33] Utazi, C. E. et al. Mapping vaccination coverage to explore the effects of delivery mechanisms and inform vaccination strategies. *Nat. Commun.* **10**, 1633. 10.1038/s41467-019-09611-1 (2019). 30967543 10.1038/s41467-019-09611-1 PMC6456602

[34] Cata-Preta, B. O. et al. Inequalities in ownership and availability of home-based vaccination records in 82 low- and middle-income countries. *BMJ Glob. Health.* **9**, https://doi.org/10.1136/bmjgh-2024-016054 (2024). 10.1136/bmjgh-2024-016054 PMC11683979 39732475

[35] WHO. *Measles-containing-vaccine second-dose (MCV2) Immunization Coverage by the Nationally Recommended Age (%) (WUENIC)*, https://www.who.int/data/gho/indicator-metadata-registry/imr-details/4756#:~:text=Definition%3 A,to%20the%20nationally%20recommended%20schedule>.

[36] DHS. *DEMOGRAPHIC AND HEALTH SURVEYS MODEL WOMAN’S QUESTIONNAIRE*, https://dhsprogram.com/pubs/pdf/DHSQ8/DHS8_Womans_QRE_EN_14Feb2023_DHSQ8.pdf

[37] NBS & UNICEF. *Multiple Indicator Cluster Survey 2021, Survey Findings Report* (Abuja, 2022).

[38] WHO. *Introduction of Measles-containing vaccine 2nd dose*, https://immunizationdata.who.int/pages/vaccine-intro-by-antigen/mcv2.html?ISO_3_CODE=&YEAR=

[39] WHO. *Measles vaccination coverage*, https://immunizationdata.who.int/global/wiise-detail-page/measles-vaccination-coverage

[40] WHO. *WHO/UNICEF estimates of national immunization coverage*, https://www.who.int/teams/immunization-vaccines-and-biologicals/immunization-analysis-and-insights/global-monitoring/immunization-coverage/who-unicef-estimates-of-national-immunization-coverage

[41] Seidenberg, A. B., Moser, R. P. & West, B. T. Preferred reporting items for complex sample survey analysis (PRICSSA). *J. Surv. Stat. Methodol.* **11**, 743–757. 10.1093/jssam/smac040 (2023).

[42] Croft, T. N. et al. *Guide to DHS Statistics*. (2023).

