# Supplementary material for: Factors determining the overlap between recipients of the first and second dose of measles vaccine in nineteen surveys
Source: Sci Rep. 2025 Aug 21;15:30737. doi: 10.1038/s41598-025-10678-8 (PMC12371077; doi:10.1038/s41598-025-10678-8)
Supplement: Supplementary file 1 — Supplementary Material 1 [file 41598_2025_10678_MOESM1_ESM.pdf]

Factors determining the overlap between recipients of the first and second dose of measles vaccine in 19 surveys – Supplementary material

Section 1

Table S1: Vaccination status among 24-35 month olds in each survey. Numbers presented are weighted counts rounded to integer values. Due to that in some cases break down columns do not sum up exactly to 'Total' values. Surveys for which MCV2 was introduced less than 3 years before the survey are denoted with an asterisk. Columns 6 and 11 hold WUENIC coverage estimates for the year in which each child was aged 1 year (for MCV1) or 2 years (for MCV2), i.e. respectively 2 and 1 years before the year or years of the survey. For the surveys which spanned more than one year, the WUENIC coverage for the given vaccine is provided for each of the possible years in which children were aged 1 or 2 years and the values are separated by a comma.

Country surveyed, year	Number of children aged 24-35 months	Number (%) of children whose measles 1 vaccination status was			WUENIC MCV1 coverage estimate for corresponding year(s)	Number (%) of children whose measles 2 vaccination status was			WUENIC MCV2 coverage estimate for corresponding year(s)	Year of MCV2 intro-duction
		Unvaccinated	Probable	Don't know		Unvaccinated	Probable	Don't know		
Afghanistan 2015	6570	2514 (38.3%)	3916 (59.6%)	139 (2.1%)	57%	3880 (59.1%)	2551 (38.8%)	139 (2.1%)	44%	2004
Angola 2015-16*	2495	980 (39.3%)	1460 (58.5%)	55 (2.2%)	59%,56%	1776 (71.2%)	665 (26.6%)	55 (2.2%)	NA,16%	2015
Bangladesh 2017-18	1685	123 (7.3%)	1558 (92.5%)	4 (0.2%)	97%,97%	276 (16.4%)	1405 (83.4%)	4 (0.2%)	92%,93%	2012
Burundi 2016-17	2443	107 (4.4%)	2336 (95.6%)	0 (0%)	94%,93%	582 (23.8%)	1861 (76.2%)	0 (0%)	65%,72%	2013
Jordan 2017-18	1891	194 (10.2%)	1693 (89.5%)	5 (0.3%)	94%,96%	311 (16.5%)	1575 (83.3%)	5 (0.3%)	99%,99%	1995
Malawi 2015-16*	3261	212 (6.5%)	3043 (93.3%)	7 (0.2%)	88%,85%	2673 (82%)	581 (17.8%)	7 (0.2%)	NA,8%	2015
Maldives 2016-17	512	32 (6.3%)	469 (91.7%)	10 (2%)	99%,99%	116 (22.7%)	386 (75.3%)	10 (2%)	99%,99%	2007
Myanmar 2015-16	782	122 (15.6%)	658 (84.1%)	2 (0.2%)	86%,88%	291 (37.3%)	489 (62.5%)	2 (0.2%)	82%,78%	2012
Nigeria 2018*	5835	2460 (42.2%)	3361 (57.6%)	14 (0.2%)	48%	4906 (84.1%)	915 (15.7%)	14 (0.2%)	NA	2020
Pakistan 2017-18	1919	452 (23.6%)	1444 (75.3%)	22 (1.2%)	75%,75%	617 (32.2%)	1279 (66.7%)	22 (1.2%)	67%,67%	2009
Papua New Guinea 2016-18*	1828	669 (36.6%)	1112 (60.8%)	47 (2.6%)	65%,57%,46%	924 (50.6%)	857 (46.9%)	47 (2.6%)	NA,NA,NA	2016
Philippines 2017	1835	339 (18.5%)	1478 (80.5%)	18 (1%)	80%	958 (52.2%)	858 (46.8%)	18 (1%)	62%	2010
Senegal 2017	2102	203 (9.6%)	1892 (90%)	7 (0.4%)	80%	1016 (48.3%)	1079 (51.3%)	7 (0.4%)	57%	2014
Senegal 2018	1092	103 (9.4%)	989 (90.6%)	0 (0%)	93%	425 (38.9%)	667 (61.1%)	0 (0%)	59%	2014
Senegal 2019	1147	113 (9.9%)	1030 (89.9%)	3 (0.3%)	90%	431 (37.6%)	712 (62.1%)	3 (0.3%)	62%	2014
Sierra Leone 2019	1666	290 (17.4%)	1369 (82.2%)	6 (0.4%)	80%	743 (44.6%)	917 (55%)	6 (0.4%)	64%	2015
South Africa 2016	660	65 (9.8%)	556 (84.2%)	40 (6.1%)	84%	227 (34.4%)	393 (59.5%)	40 (6.1%)	59%	1994
Tanzania 2015-16*	1817	175 (9.6%)	1639 (90.2%)	2 (0.1%)	99%,97%	1239 (68.2%)	576 (31.7%)	2 (0.1%)	29%,53%	2014
Zambia 2018	1862	126 (6.8%)	1734 (93.1%)	2 (0.1%)	97%	672 (36.1%)	1188 (63.8%)	2 (0.1%)	64%	2013

Section 2

Table S2: Description of the source of data on measles 1 and measles 2 vaccination status among children aged 24-35 months. Numbers presented are weighted counts rounded to integer values. Due to that in some cases break down columns do not sum up exactly to 'Total' values. Surveys for which MCV2 was introduced less than 3 years before the survey are denoted with an asterisk.

Country surveyed, year	Number with probable measles 1 vaccination	Number (%) with probable measles 1 vaccination whose measles 1 vaccination status was based on:			Number with probable measles 2 vaccination	Number (%) with probable measles 2 vaccination whose measles2 vaccination status was based on:		
		Mother's report	Vaccination card with date	Vaccination card with no date		Mother's report	Vaccination card with date	Vaccination card with no date
Afghanistan 2015	3916	2076 (53%)	1806 (46.1%)	35 (0.9%)	2551	1326 (52%)	1192 (46.7%)	33 (1.3%)
Angola 2015-16*	1460	866 (59.3%)	571 (39.1%)	23 (1.6%)	665	360 (54.2%)	255 (38.3%)	50 (7.5%)
Bangladesh 2017-18	1558	494 (31.7%)	1058 (67.9%)	7 (0.4%)	1405	445 (31.7%)	947 (67.4%)	12 (0.9%)
Burundi 2016-17	2336	962 (41.2%)	1356 (58.1%)	18 (0.7%)	1861	971 (52.2%)	862 (46.3%)	28 (1.5%)
Jordan 2017-18	1693	451 (26.6%)	1202 (71%)	40 (2.4%)	1575	368 (23.3%)	1157 (73.5%)	50 (3.2%)
Malawi 2015-16*	3043	1136 (37.3%)	1814 (59.6%)	93 (3%)	581	467 (80.3%)	53 (9.1%)	62 (10.6%)
Maldives 2016-17	469	76 (16.2%)	380 (81.1%)	13 (2.8%)	386	20 (5.1%)	353 (91.5%)	13 (3.4%)
Myanmar 2015-16	658	412 (62.6%)	245 (37.2%)	1 (0.1%)	489	312 (63.9%)	174 (35.7%)	2 (0.4%)
Nigeria 2018*	3361	2127 (63.3%)	1132 (33.7%)	102 (3%)	915	633 (69.2%)	224 (24.5%)	58 (6.3%)
Pakistan 2017-18	1444	654 (45.3%)	788 (54.6%)	2 (0.1%)	1279	593 (46.3%)	680 (53.1%)	7 (0.5%)
Papua New Guinea 2016-18*	1112	346 (31.2%)	704 (63.3%)	61 (5.5%)	857	233 (27.2%)	549 (64%)	75 (8.7%)
Philippines 2017	1478	572 (38.7%)	905 (61.2%)	1 (0.1%)	858	243 (28.3%)	613 (71.4%)	2 (0.3%)
Senegal 2017	1892	735 (38.8%)	1128 (59.6%)	30 (1.6%)	1079	297 (27.5%)	766 (71%)	16 (1.5%)
Senegal 2018	989	374 (37.9%)	602 (60.9%)	12 (1.2%)	667	239 (35.8%)	414 (62%)	15 (2.2%)
Senegal 2019	1030	350 (34%)	669 (64.9%)	12 (1.1%)	712	205 (28.8%)	498 (69.9%)	9 (1.3%)
Sierra Leone 2019	1369	568 (41.5%)	761 (55.6%)	41 (3%)	917	380 (41.4%)	502 (54.8%)	35 (3.8%)
South Africa 2016	556	173 (31.2%)	305 (55%)	77 (13.8%)	393	74 (18.7%)	247 (62.9%)	72 (18.4%)
Tanzania 2015-16*	1639	477 (29.1%)	1138 (69.4%)	25 (1.5%)	576	241 (41.8%)	297 (51.5%)	39 (6.7%)
Zambia 2018	1734	580 (33.5%)	1117 (64.4%)	37 (2.1%)	1188	402 (33.8%)	763 (64.2%)	24 (2%)

Section 3

Table S3: MCV1 and MCV2 status among the children aged 24-35 months whose vaccination status was known. Numbers presented are weighted counts rounded to integer values. Surveys for which MCV2 was introduced less than 3 years before the survey are denoted with an asterisk.

Country surveyed, year	Number (%, 95% CI) with probable MCV1 only	Number (%, 95% CI) with probable MCV2 only	Number (%, 95% CI) with neither MCV1 or MCV2	Number of children aged 24-35 months with known vaccination status
Afghanistan 2015	1399 (21.8%, 20%-23.6%)	34 (0.5%, 0.3%-0.9%)	2481 (38.6%, 36.5%-40.7%)	6431
Angola 2015-16*	801 (32.8%, 30.3%-35.4%)	5 (0.2%, 0.1%-0.5%)	975 (40%, 37.5%-42.5%)	2441
Bangladesh 2017-18	158 (9.4%, 7.9%-11.1%)	4 (0.2%, 0.1%-0.8%)	118 (7%, 5.8%-8.5%)	1681
Burundi 2016-17	487 (19.9%, 18.3%-21.7%)	11 (0.5%, 0.3%-0.9%)	95 (3.9%, 3.1%-4.8%)	2443
Jordan 2017-18	120 (6.4%, 5%-8%)	2 (0.1%, 0%-0.3%)	191 (10.1%, 8.3%-12.4%)	1886
Malawi 2015-16*	2465 (75.8%, 73.9%-77.5%)	4 (0.1%, 0%-0.4%)	208 (6.4%, 5.4%-7.5%)	3254
Maldives 2016-17	84 (16.7%, 12.8%-21.4%)	0 (0%, 0%-0%)	32 (6.5%, 4.8%-8.6%)	502
Myanmar 2015-16	171 (22%, 18.8%-25.5%)	2 (0.3%, 0.1%-1.1%)	120 (15.4%, 12.7%-18.5%)	780
Nigeria 2018*	2453 (42.1%, 40.7%-43.6%)	7 (0.1%, 0.1%-0.2%)	2453 (42.1%, 40.7%-43.6%)	5821
Pakistan 2017-18	165 (8.7%, 7.2%-10.5%)	1 (0%, 0%-0%)	452 (23.8%, 21.4%-26.4%)	1897
Papua New Guinea 2016-18*	274 (15.4%, 13.1%-17.9%)	19 (1%, 0.7%-1.6%)	651 (36.5%, 33.6%-39.6%)	1781
Philippines 2017	619 (34.1%, 31.2%-37.1%)	0 (0%, 0%-0%)	339 (18.7%, 16.4%-21.2%)	1817
Senegal 2017	820 (39.1%, 36.7%-41.7%)	7 (0.3%, 0.2%-0.6%)	196 (9.4%, 8.1%-10.8%)	2095
Senegal 2018	324 (29.7%, 26.4%-33.2%)	3 (0.2%, 0.1%-0.7%)	100 (9.2%, 7.4%-11.5%)	1092
Senegal 2019	326 (28.5%, 25.3%-31.9%)	8 (0.7%, 0.3%-1.4%)	105 (9.2%, 7.5%-11.3%)	1143
Sierra Leone 2019	463 (27.9%, 25.6%-30.3%)	11 (0.7%, 0.3%-1.4%)	279 (16.8%, 14.9%-18.9%)	1660
South Africa 2016	165 (26.5%, 22.4%-31.2%)	2 (0.3%, 0.1%-1.4%)	63 (10.1%, 7.2%-14%)	620
Tanzania 2015-16*	1067 (58.8%, 56%-61.5%)	3 (0.2%, 0%-0.7%)	172 (9.5%, 8%-11.2%)	1815
Zambia 2018	546 (29.4%, 26.8%-32.1%)	0 (0%, 0%-0%)	126 (6.8%, 5.4%-8.5%)	1860

Section 4

Table S4: Type and age of scheduled routine measles containing vaccinations for the countries included in the analysis as they are reported by WHO at the time of this publication. The year of MCV2 introduction for each country is listed in Table S13. WHO reports are based on reports provided by the country and UNICEF in the Joint Reporting Form [1,2]

Country	Scheduled measles-containing vaccination		
Afghanistan	Measles 9 months	Measles 18 months	
Angola	MR 9 months	MR 15 months	
Bangladesh	MR 9 months	MR 15 months	
Burundi	MR 9 months	MR 18 months	
Jordan	Measles 9 months	MMR 12 months	MMR 18 months
Malawi	MR 9 months	MR 15 months	
Maldives	MR 9 months	MMR 18 months	
Myanmar	MR 9 months	MR 18 months	
Nigeria	MR 9 months	MMR 18 months	
Pakistan	MR 9 months	MR 15 months	
Papua New Guinea	MR 9 months	MR 18 months	MR 7 years
Philippines	MMR 9 months	MMR 12-15 months	MR 5-6 & 12-13 years
Senegal	MR 9 months	MR 15 months	
Sierra Leone	MR 9 months	MR 15 months	
South Africa	Measles 6 months	Measles 12 months	
Tanzania	MR 9 months	MR 18 months	
Zambia	MR 9 months	MR 18 months	

Section 5

Table S5: Percentage of children with probable MCV1 who also received MCV2 and of children with probable MCV2 who had also received MCV1, calculated using children whose vaccination status was determined either from the vaccination card or from the mother's report. Numbers presented are weighted counts rounded to integer values. Surveys for which MCV2 was introduced less than 3 years before the survey are denoted with an asterisk.

Country surveyed, year	Number (%, 95% CI) of children with probable MCV1 whose MCV2 status was probable	Number of children with probable MCV1	Number (%, 95% CI) of children with probable MCV2 whose MCV1 status was probable	Number of children with probable MCV2
Afghanistan 2015	2517 (64.3%, 61.5%-66.9%)	3916	2517 (98.7%, 97.7%-99.2%)	2551
Angola 2015-16*	660 (45.2%, 41.5%-48.8%)	1460	660 (99.2%, 98%-99.7%)	665
Bangladesh 2017-18	1400 (89.9%, 88%-91.5%)	1558	1400 (99.7%, 99%-99.9%)	1405
Burundi 2016-17	1849 (79.2%, 77.3%-80.9%)	2336	1849 (99.4%, 98.8%-99.6%)	1861
Jordan 2017-18	1573 (92.9%, 91.1%-94.4%)	1693	1573 (99.9%, 99.7%-99.9%)	1575
Malawi 2015-16*	577 (19%, 17.3%-20.8%)	3043	577 (99.3%, 97.8%-99.8%)	581
Maldives 2016-17	386 (82.2%, 77.2%-86.3%)	469	386 (100%, 100%-100%)	386
Myanmar 2015-16	486 (73.9%, 69.9%-77.6%)	658	486 (99.5%, 98.3%-99.9%)	489
Nigeria 2018*	908 (27%, 25.3%-28.8%)	3361	908 (99.2%, 98.4%-99.6%)	915
Pakistan 2017-18	1279 (88.5%, 86.2%-90.5%)	1444	1279 (100%, 99.8%-100%)	1279
Papua New Guinea 2016-18*	838 (75.4%, 71.7%-78.8%)	1112	838 (97.8%, 96.6%-98.6%)	857
Philippines 2017	858 (58.1%, 54.6%-61.5%)	1478	858 (100%, 100%-100%)	858
Senegal 2017	1073 (56.7%, 54%-59.4%)	1892	1073 (99.4%, 98.8%-99.7%)	1079
Senegal 2018	665 (67.2%, 63.5%-70.8%)	989	665 (99.6%, 98.9%-99.9%)	667
Senegal 2019	705 (68.4%, 64.6%-71.9%)	1030	705 (98.9%, 97.7%-99.5%)	712
Sierra Leone 2019	906 (66.2%, 63.4%-68.8%)	1369	906 (98.8%, 97.5%-99.4%)	917
South Africa 2016	391 (70.4%, 65.4%-74.9%)	556	391 (99.5%, 97.8%-99.9%)	393
Tanzania 2015-16*	573 (34.9%, 32.2%-37.8%)	1639	573 (99.4%, 97.8%-99.9%)	576
Zambia 2018	1188 (68.5%, 65.6%-71.2%)	1734	1188 (100%, 100%-100%)	1188

Section 6

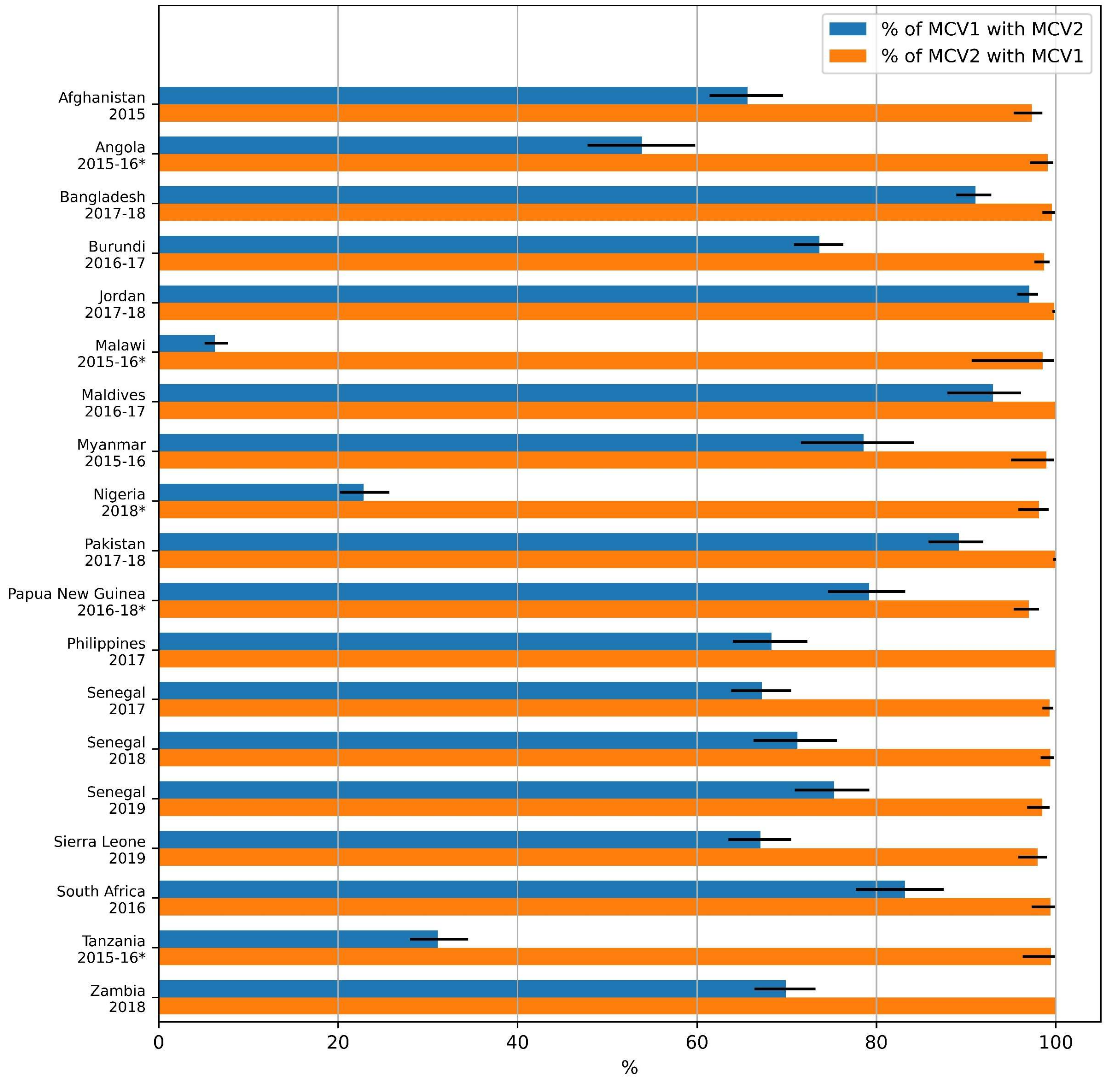

Figure S1: Percentage of children with probable MCV1 who also received MCV2 and of children with probable MCV2 who had also received MCV1 including only the cases noted on vaccination card. The numerical data depicted in the figure are also included in tabular form in Table S6. Surveys for which MCV2 was introduced less than 3 years before the survey are denoted with an asterisk. As is described in the main paper, instances of children with MCV2 but not MCV1 should be attributed to errors in the data collection or data cleanup,

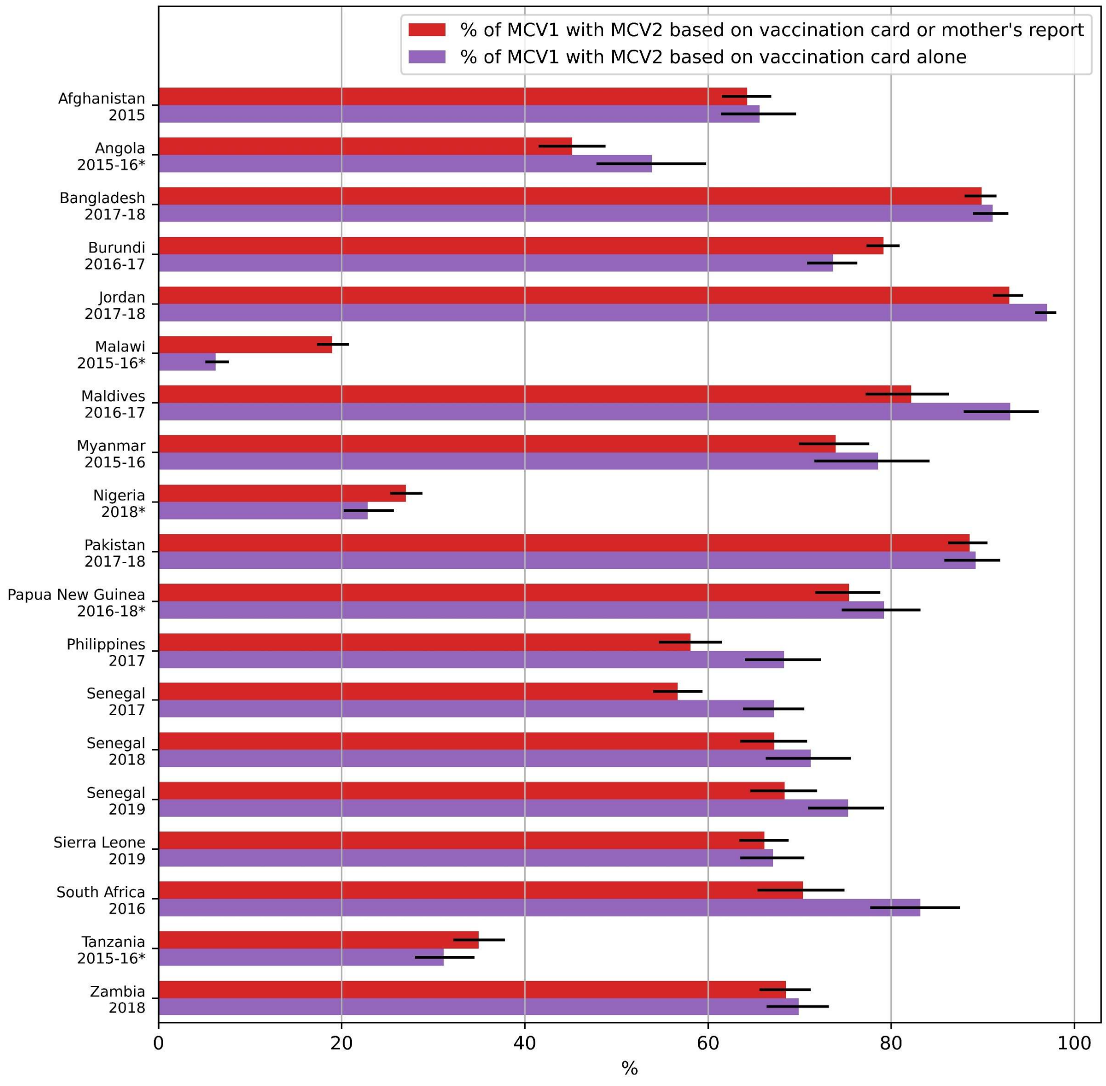

Figure S2: Comparison between the percentage of children with probable MCV1 who also received MCV2 as calculated by including only the children whose vaccination status was determined from the vaccination card, with or without the mother's report, against that obtained by including children whose vaccination status was determined from the vaccination card alone. The numerical data depicted in the figure are also included in tabular form in the supplement (see Tables S5 and S6). Surveys for which MCV2 was introduced less than 3 years before the survey are denoted with an asterisk.

Table S6: Percentage of children with probable MCV1 who also received MCV2 and of children with probable MCV2 who had also received MCV1 based on children whose vaccination status was determined by the vaccination card alone. Numbers presented are weighted counts rounded to integer values. Surveys for which MCV2 was introduced less than 3 years before the survey are denoted with an asterisk. Column 4 replicates column 2 of Table S5 for easy comparison with the results obtained based on the mother's report or the vaccination card.

Country surveyed, year	Number (%, 95% CI) of children with MCV1 (based on vaccination card) who subsequently had MCV2 (based on vaccination card)	Number of children with MCV1 (based on vaccination card)	Number (%, 95% CI) of children with probable MCV1 whose MCV2 status was probable based on either the vaccination card or mother's report (copied from Table S5)	Number (%, 95% CI) of children with MCV2 (based on vaccination card) who previously had MCV1 (based on vaccination card)	Number of children with MCV2 (based on vaccination card)
Afghanistan 2015	1190 (65.6%, 61.4%-69.6%)	1813	2517 (64.3%, 61.5%-66.9%)	1190 (97.3%, 95.3%-98.5%)	1222
Angola 2015-16*	300 (53.9%, 47.8%-59.8%)	558	660 (45.2%, 41.5%-48.8%)	300 (99.1%, 97.1%-99.7%)	303
Bangladesh 2017-18	955 (91.1%, 88.9%-92.8%)	1049	1400 (89.9%, 88%-91.5%)	955 (99.6%, 98.5%-99.9%)	959
Burundi 2016-17	863 (73.6%, 70.8%-76.3%)	1171	1849 (79.2%, 77.3%-80.9%)	863 (98.7%, 97.6%-99.3%)	874
Jordan 2017-18	1205 (97.0%, 95.7%-98.0%)	1242	1573 (92.9%, 91.1%-94.4%)	1205 (99.8%, 99.6%-99.9%)	1207
Malawi 2015-16*	110 (6.2%, 5.1%-7.7%)	1763	577 (19%, 17.3%-20.8%)	110 (98.5%, 90.6%-99.8%)	112
Maldives 2016-17	366 (93.0%, 87.9%-96.1%)	393	386 (82.2%, 77.2%-86.3%)	366 (100.0%, 100.0%-100.0%)	366
Myanmar 2015-16	173 (78.6%, 71.6%-84.2%)	220	486 (73.9%, 69.9%-77.6%)	173 (98.9%, 95.0%-99.8%)	175
Nigeria 2018*	274 (22.8%, 20.2%-25.7%)	1201	908 (27%, 25.3%-28.8%)	274 (98.1%, 95.8%-99.2%)	280
Pakistan 2017-18	683 (89.2%, 85.8%-91.9%)	765	1279 (88.5%, 86.2%-90.5%)	683 (99.9%, 99.7%-100.0%)	683
Papua New Guinea 2016-18*	599 (79.2%, 74.6%-83.2%)	757	838 (75.4%, 71.7%-78.8%)	599 (97.0%, 95.3%-98.1%)	618
Philippines 2017	615 (68.3%, 64.0%-72.3%)	901	858 (58.1%, 54.6%-61.5%)	615 (100.0%, 100.0%-100.0%)	615
Senegal 2017	769 (67.2%, 63.8%-70.5%)	1144	1073 (56.7%, 54%-59.4%)	769 (99.3%, 98.5%-99.7%)	774
Senegal 2018	423 (71.2%, 66.3%-75.6%)	595	665 (67.2%, 63.5%-70.8%)	423 (99.4%, 98.3%-99.8%)	426
Senegal 2019	499 (75.3%, 70.9%-79.2%)	663	705 (68.4%, 64.6%-71.9%)	499 (98.5%, 96.8%-99.3%)	506
Sierra Leone 2019	526 (67.1%, 63.5%-70.5%)	785	906 (66.2%, 63.4%-68.8%)	526 (98.0%, 95.8%-99.0%)	537
South Africa 2016	317 (83.2%, 77.7%-87.5%)	381	391 (70.4%, 65.4%-74.9%)	317 (99.4%, 97.3%-99.9%)	319
Tanzania 2015-16*	332 (31.1%, 28.0%-34.5%)	1067	573 (34.9%, 32.2%-37.8%)	332 (99.5%, 96.3%-99.9%)	334
Zambia 2018	778 (69.9%, 66.4%-73.2%)	1114	1188 (68.5%, 65.6%-71.2%)	778 (100.0%, 100.0%-100.0%)	778

Section 7

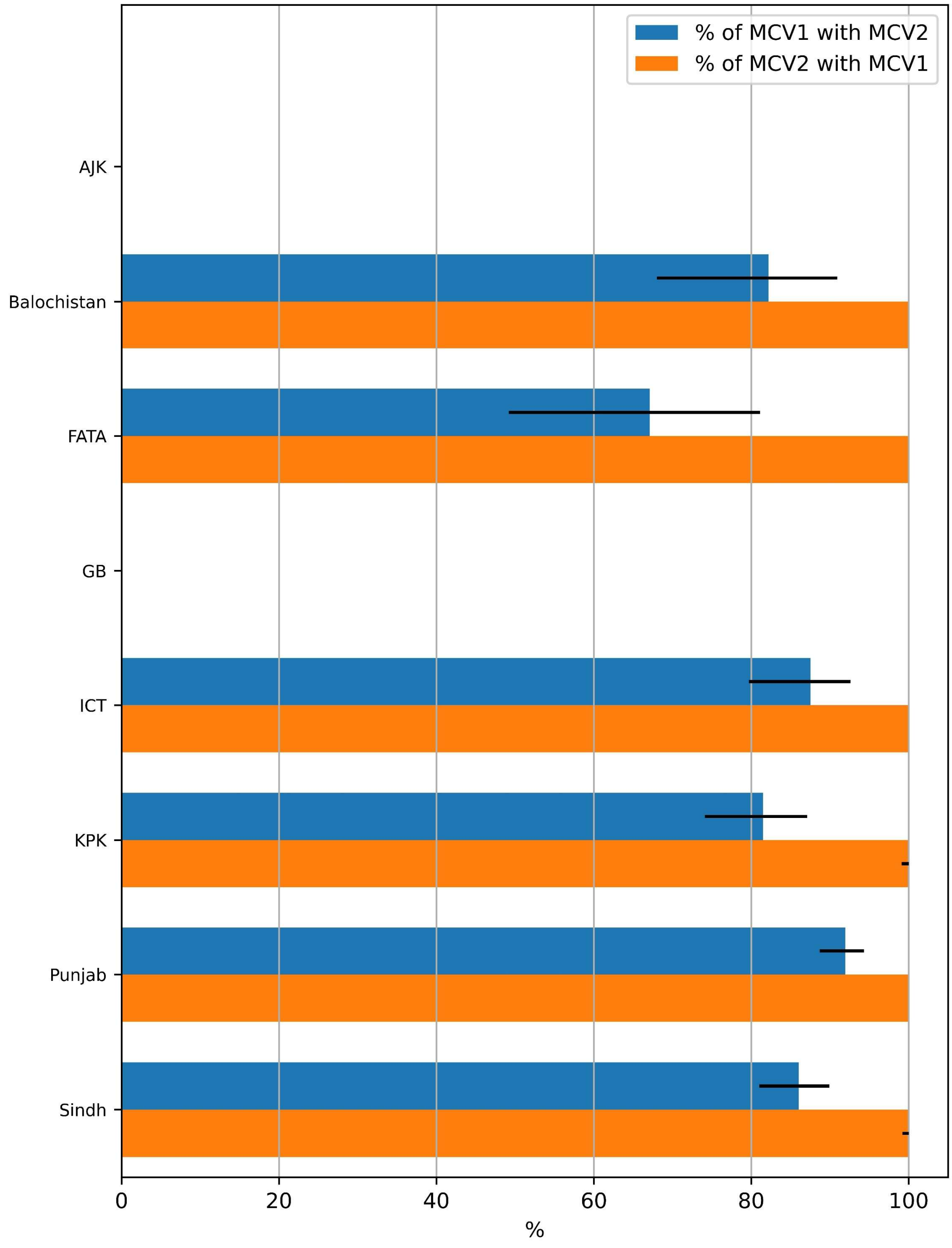

Figure S3: Percentage of children with probable MCV1 who also probably received MCV2 and of children with probable MCV2 who had also probably received MCV1 for the 8 ADM1 level provinces of Pakistan. The application of DHS survey data sampling weighting did not return usable results for the two provinces of AJK and GB. As is described in the main paper, instances of children with MCV2 but not MCV1 should be attributed to errors in the data collection or data cleanup,

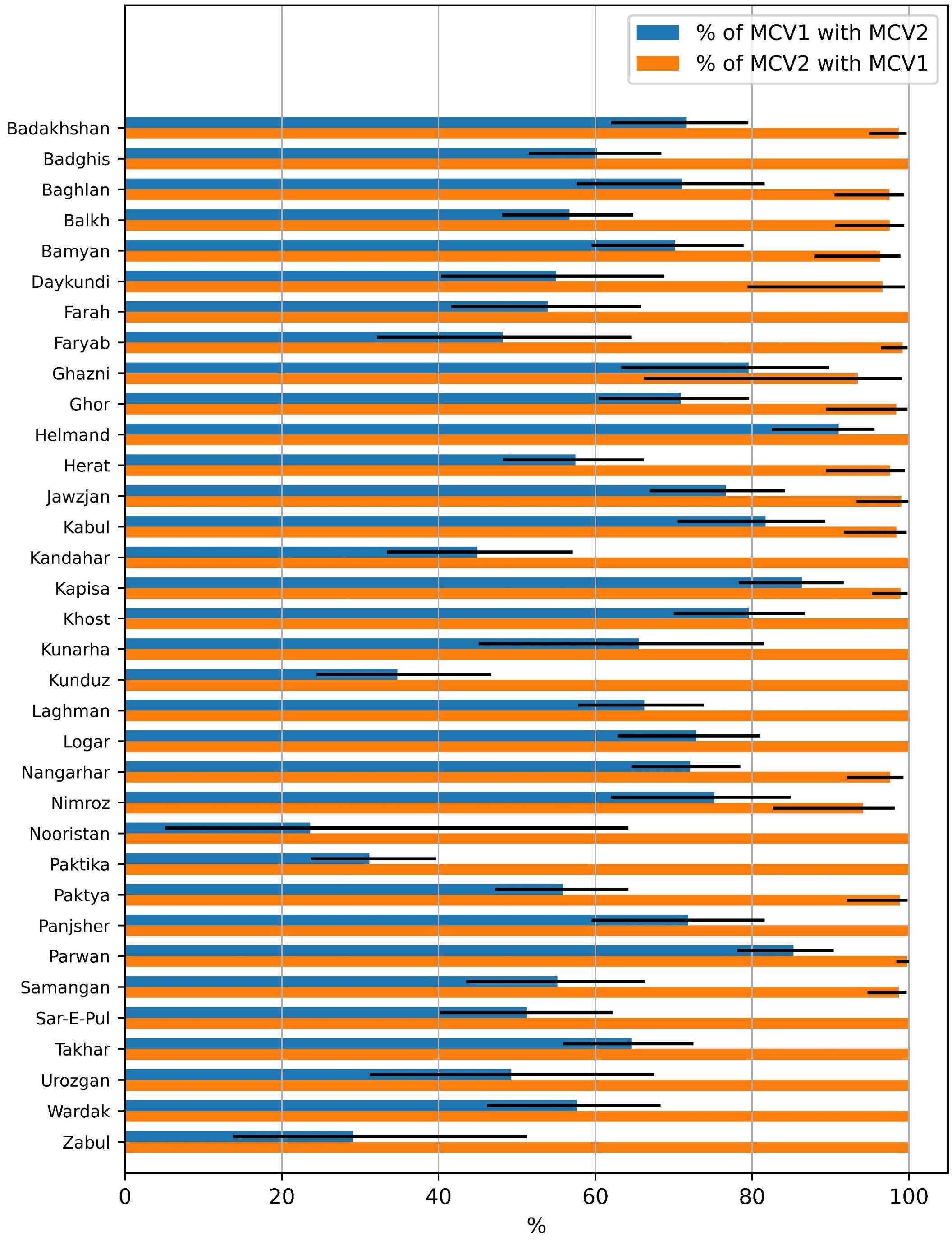

Figure S4: Percentage of children with probable MCV1 who also probably received MCV2 and of children with probable MCV2 who had also probably received MCV1 for the 34 ADM1 level provinces of Afghanistan. As is described in the main paper, instances of children with MCV2 but not MCV1 should be attributed to errors in the data collection or data cleanup,

Table S7: Percentage of children with probable MCV1 who also received MCV2 and of children with probable MCV2 who had also received MCV1 for ADM1 level provinces of Pakistan 2017-18. The application of DHS survey data sampling weighting did not return usable results for the two regions of AJK and GB

Region	Number (%, 95% CI) of children with probable MCV1 whose MCV2 status was probable	Number of children with probable MCV1	Number (%, 95% CI) of children with probable MCV2 whose MCV1 status was probable	Number of children with probable MCV2	Number of children aged 24-35 months with known vaccination status
AJK					
Balochistan	36 (82.2%, 68.0%-90.9%)	44	36 (100.0%, 100%-100%)	36	98
FATA	11 (67.1%, 49.2%-81.1%)	16	11 (100.0%, 100%-100%)	11	53
GB					
ICT	11 (87.5%, 79.7%-92.6%)	12	11 (100.0%, 100%-100%)	11	14
KPK	168 (81.5%, 74.1%-87.1%)	206	168 (99.9%, 99.1%-100.0%)	168	334
Punjab	785 (91.9%, 88.7%-94.3%)	854	785 (100.0%, 100%-100%)	785	958
Sindh	268 (86.0%, 81.0%-89.9%)	311	268 (99.9%, 99.2%-100.0%)	268	440

Table S8: Percentage of children with probable MCV1 who also received MCV2 and of children with probable MCV2 who had also received MCV1 for ADM1 level provinces of Afghanistan 2015. Cases where the number of children appears as equal to zero but the percentage and 95% confidence interval is non-zero are due to rounding effects after the application of weighting according to the DHS stratified sampling design

Region	Number (%, 95% CI) of children with probable MCV1 whose MCV2 status was probable	Number of children with probable MCV1	Number (%, 95% CI) of children with probable MCV2 whose MCV1 status was probable	Number of children with probable MCV2	Number of children aged 24-35 months with known vaccination status
Badakhshan	136 (71.6%, 62.0%-79.5%)	190	136 (98.7%, 94.9%-99.7%)	138	213
Badghis	85 (60.2%, 51.5%-68.4%)	141	85 (100.0%, 100%-100%)	85	162
Baghlan	55 (71.1%, 57.6%-81.6%)	77	55 (97.5%, 90.5%-99.4%)	56	159
Balkh	166 (56.7%, 48.1%-64.8%)	292	166 (97.5%, 90.6%-99.4%)	170	419
Bamyan	35 (70.1%, 59.5%-78.9%)	50	35 (96.3%, 87.9%-98.9%)	36	65
Daykundi	15 (55.0%, 40.3%-68.8%)	27	15 (96.6%, 79.4%-99.5%)	15	59
Farah	40 (53.9%, 41.6%-65.8%)	75	40 (100.0%, 100%-100%)	40	203
Faryab	150 (48.2%, 32.1%-64.6%)	312	150 (99.2%, 96.4%-99.8%)	152	433
Ghazni	52 (79.6%, 63.3%-89.8%)	66	52 (93.5%, 66.2%-99.1%)	56	150
Ghor	64 (70.9%, 60.4%-79.6%)	90	64 (98.4%, 89.4%-99.8%)	65	205
Helmand	80 (91.0%, 82.5%-95.6%)	88	80 (100.0%, 100%-100%)	80	184
Herat	194 (57.5%, 48.2%-66.2%)	338	194 (97.6%, 89.4%-99.5%)	199	461
Jawzjan	56 (76.6%, 66.9%-84.2%)	73	56 (99.0%, 93.3%-99.9%)	56	165
Kabul	477 (81.7%, 70.5%-89.3%)	583	477 (98.4%, 91.7%-99.7%)	484	783
Kandahar	62 (44.9%, 33.4%-57.1%)	139	62 (100.0%, 100%-100%)	62	573
Kapisa	25 (86.3%, 78.3%-91.7%)	29	25 (98.9%, 95.3%-99.8%)	25	41
Khost	74 (79.6%, 70.0%-86.7%)	93	74 (100.0%, 100%-100%)	74	234
Kunarha	33 (65.5%, 45.1%-81.5%)	50	33 (100.0%, 100%-100%)	33	123
Kunduz	46 (34.7%, 24.4%-46.7%)	134	46 (100.0%, 100%-100%)	46	249
Laghman	96 (66.2%, 57.8%-73.8%)	144	96 (100.0%, 100%-100%)	96	175
Logar	38 (72.8%, 62.8%-81.0%)	52	38 (100.0%, 100%-100%)	38	87
Nangarhar	92 (72.1%, 64.6%-78.5%)	128	92 (97.6%, 92.1%-99.3%)	94	167
Nimroz	25 (75.2%, 62.0%-84.9%)	34	25 (94.1%, 82.6%-98.2%)	27	51
Nooristan	0 (23.6%, 5.1%-64.2%)	2	0 (100.0%, 100%-100%)	0	66
Paktika	29 (31.1%, 23.7%-39.7%)	93	29 (100.0%, 100%-100%)	29	134
Paktya	60 (55.9%, 47.2%-64.2%)	108	60 (98.8%, 92.1%-99.8%)	61	137
Panjsher	4 (71.9%, 59.5%-81.6%)	6	4 (100.0%, 100%-100%)	4	9
Parwan	93 (85.3%, 78.1%-90.4%)	109	93 (99.8%, 98.4%-100.0%)	93	130
Samangan	24 (55.2%, 43.5%-66.3%)	44	24 (98.7%, 94.7%-99.7%)	25	73
Sar-E-Pul	44 (51.2%, 40.2%-62.2%)	85	44 (100.0%, 100%-100%)	44	134
Takhar	134 (64.6%, 55.9%-72.5%)	208	134 (100.0%, 100%-100%)	134	255
Urozgan	4 (49.3%, 31.2%-67.5%)	9	4 (100.0%, 100%-100%)	4	65
Wardak	26 (57.6%, 46.2%-68.3%)	46	26 (100.0%, 100%-100%)	26	61
Zabul	1 (29.1%, 13.8%-51.3%)	3	1 (100.0%, 100%-100%)	1	5

Section 8

Table S9: MCV1 status and proportion of children with probable MCV1 who also probably received MCV2 according to age at MCV1. Surveys for which MCV2 was introduced less than 3 years before the survey are denoted with an asterisk. For Jordan, the information reported by WHO at the time of this publication lists three MCV doses, the first one at 9 months as measles alone and the next two as MMR in the second year of life (see also information in Table S4 and Figure 3 in the main text). This schedule probably explains the low number of children with probable MCV1 by age 12 months in Jordan.

Country surveyed, year	Number of children with probable MCV1 by age 12 months	Number of children with probable MCV1 after age 12 months	Percentage of probable MCV1 recipients who probably received MCV1 by age 12 months	Number (%, 95% CI) with probable MCV1 by 12 months whose MCV2 status was probable	Number (%, 95% CI) with probable MCV1 after age 12 months whose MCV2 status was probable	Right-tailed p-value for whether the proportion of probable MCV1 recipients who went on to receive MCV2 was greater for those who received MCV1 before age 12 months than for those who received MVC1 after age 12 months
Afghanistan 2015	1419	387	78.6%	958 (67.5%, 62.8%-71.8%)	228 (58.9%, 48.6%-68.7%)	0.068
Angola 2015-16*	479	92	83.9%	281 (58.7%, 51.7%-65%)	43 (46.7%, 33.9%-60.2%)	0.0635
Bangladesh 2017-18	1006	52	95.1%	925 (91.9%, 89.9%-93.7%)	40 (76.9%, 61.5%-86.7%)	0.0085
Burundi 2016-17	1314	43	96.8%	1020 (77.6%, 75.1%-80%)	30 (69.8%, 53.3%-82.2%)	0.147
Jordan 2017-18	276	926	23.0%	275 (99.6%, 98.7%-99.9%)	892 (96.3%, 94.6%-97.5%)	<0.0001
Malawi 2015-16*	1613	200	89.0%	194 (12%, 10.2%-14.2%)	16 (8%, 4.6%-14.3%)	0.071
Maldives 2016-17	368	13	96.6%	341 (92.7%, 87.3%-96%)	12 (92.3%, 69.5%-98.4%)	0.4595
Myanmar 2015-16	230	15	93.9%	191 (83%, 76.4%-87.8%)	7 (46.7%, 23.2%-74.5%)	0.0105
Nigeria 2018*	970	161	85.8%	226 (23.3%, 20.3%-26.6%)	25 (15.5%, 10.5%-22%)	0.009
Pakistan 2017-18	681	106	86.5%	625 (91.8%, 88.4%-94.2%)	81 (76.4%, 63.4%-85%)	0.0025
Papua New Guinea 2016-18*	553	152	78.4%	469 (84.8%, 79.8%-88.7%)	79 (52%, 41.5%-63.2%)	<0.0001
Philippines 2017	721	184	79.7%	560 (77.7%, 73.2%-81.5%)	59 (32.1%, 20.8%-46.4%)	<0.0001
Senegal 2017	972	156	86.2%	681 (70.1%, 66.4%-73.6%)	78 (50%, 41%-59.4%)	<0.0001
Senegal 2018	525	78	87.1%	396 (75.4%, 70.7%-79.8%)	36 (46.2%, 31.5%-62.1%)	0.0005
Senegal 2019	597	72	89.2%	471 (78.9%, 74.5%-82.8%)	39 (54.2%, 40.4%-66.9%)	0.0005
Sierra Leone 2019	608	153	79.9%	410 (67.4%, 63.5%-71.4%)	97 (63.4%, 54.9%-70.8%)	0.171
South Africa 2016	282	23	92.5%	234 (83%, 75.8%-87.9%)	13 (56.5%, 32.1%-77.7%)	0.021
Tanzania 2015-16*	1001	137	88.0%	382 (38.2%, 34.7%-41.8%)	31 (22.6%, 15.5%-31.9%)	0.0005
Zambia 2018	991	126	88.7%	730 (73.7%, 69.9%-77.1%)	60 (47.6%, 37.7%-57.2%)	<0.0001

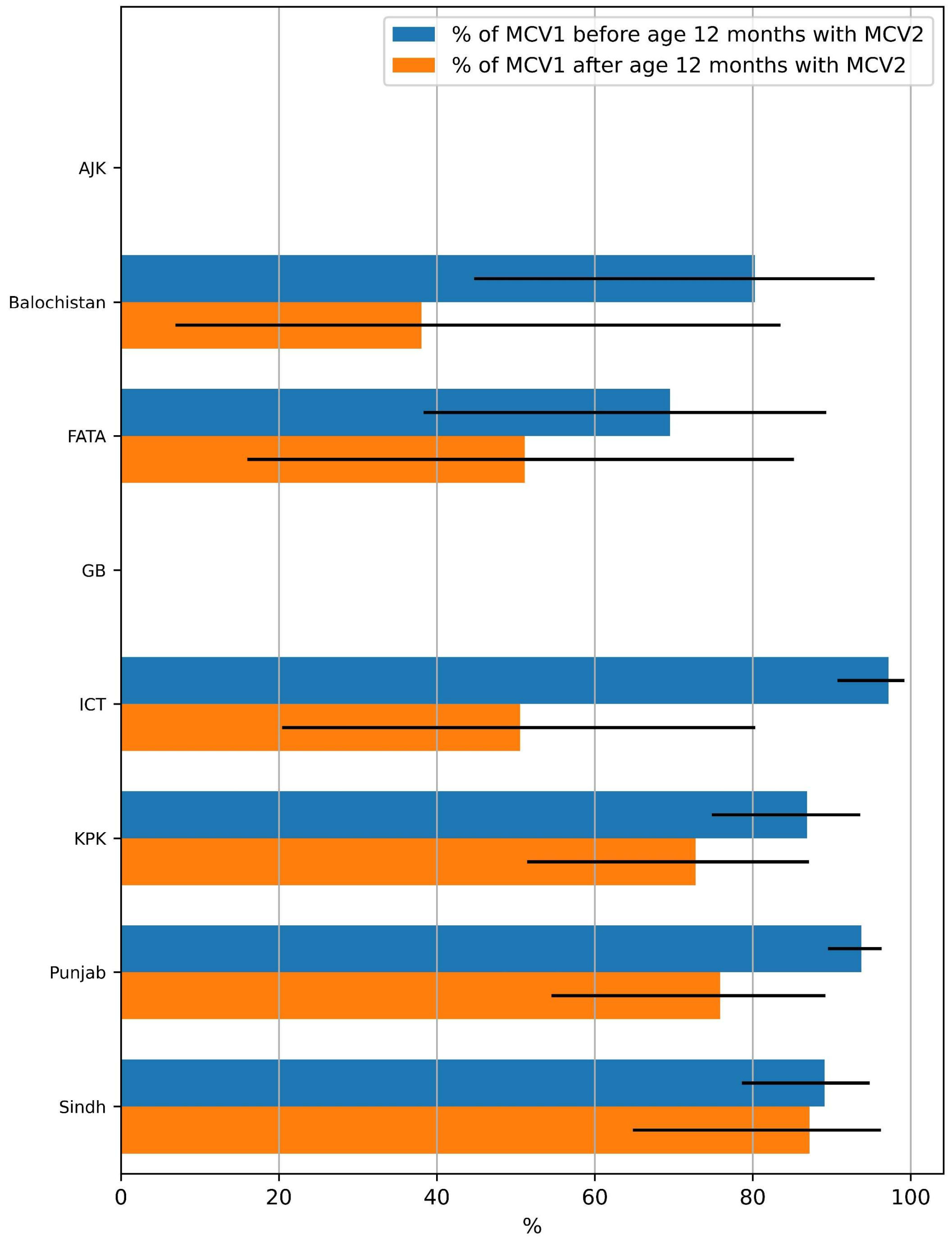

Figure S5: Percentage of children with probable MCV1 who also probably received MCV2 according to age at MCV1 for the 8 ADM1 level provinces of Pakistan. The application of DHS survey data sampling weighting did not return usable results for the two provinces of AJK and GB

Table S10: Percentage of children with probable MCV1 who also received MCV2 according to age at MCV1 for ADM1 level provinces of Pakistan. The application of DHS survey data sampling weighting did not return usable results for the two provinces of AJK and GB

Region	Number of children with probable MCV1 by age 12 months	Number of children with probable MCV1 after age 12 months	Number (%, 95% CI) with probable MCV1 by 12 months whose MCV2 status was probable	Number (%, 95% CI) with probable MCV1 after age 12 months whose MCV2 status was probable	Right-tailed p-value for whether the proportion of probable MCV1 recipients who went on to receive MCV2 was greater for those who received MCV1 before age 12 months than for those who received MVC1 after age 12 months
AJK					
Balochistan	16	1	13 (80.3%, 44.7%-95.4%)	0 (38.1%, 6.9%-83.5%)	0.077
FATA	6	3	4 (69.5%, 38.3%-89.3%)	1 (51.1%, 16%-85.2%)	0.245
GB					
ICT	6	1	6 (97.2%, 90.7%-99.2%)	1 (50.6%, 20.4%-80.3%)	0.006
KPK	77	29	67 (86.9%, 74.8%-93.6%)	21 (72.8%, 51.4%-87.1%)	0.091
Punjab	477	55	447 (93.7%, 89.5%-96.3%)	42 (75.9%, 54.5%-89.2%)	0.026
Sindh	99	18	89 (89.1%, 78.6%-94.8%)	16 (87.2%, 64.8%-96.2%)	0.412

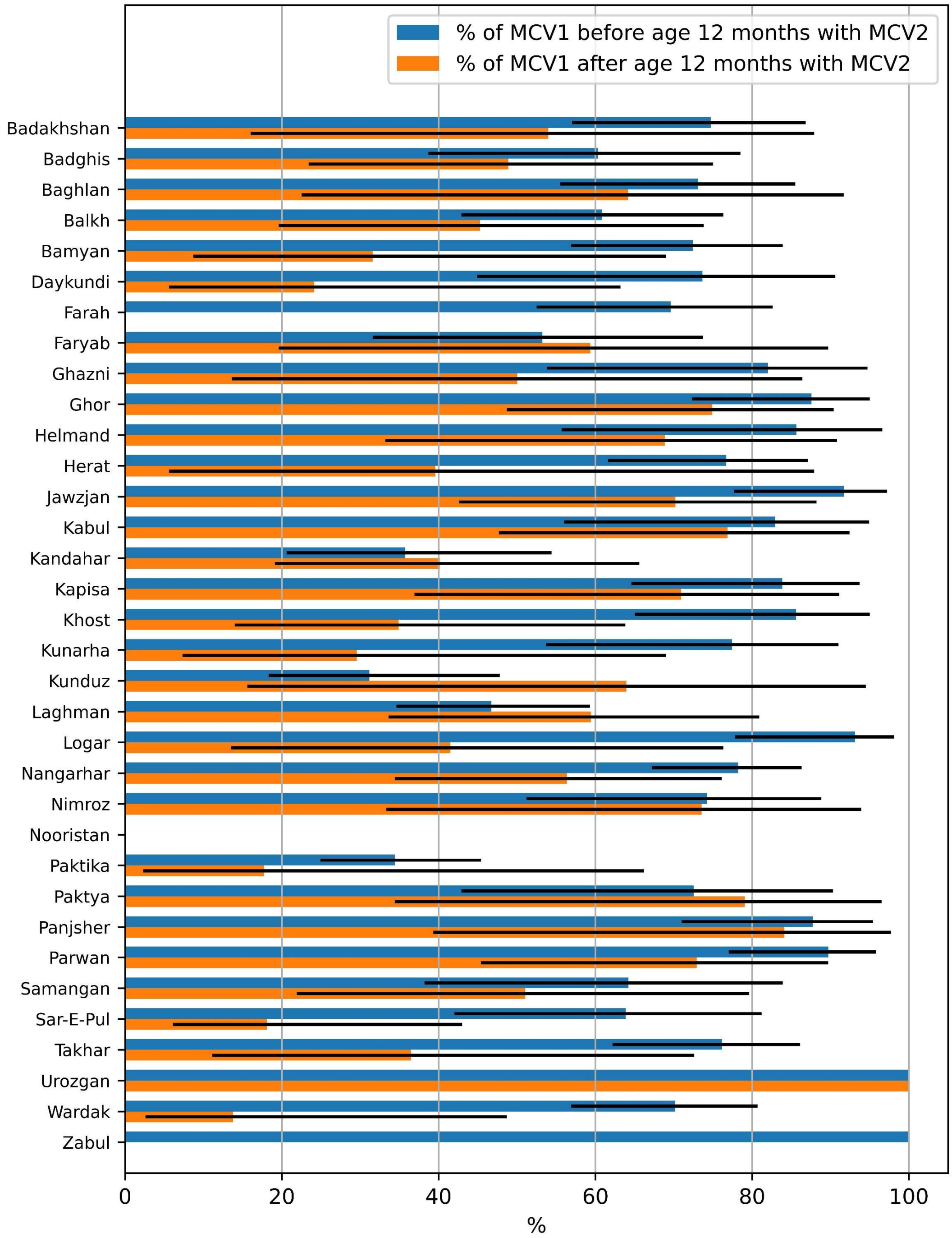

Figure S6: MCV1 status and percentage of children with probable MCV1 who also probably received MCV2 according to age at MCV1 for the 34 ADM1 level provinces of Afghanistan. The application of DHS survey data sampling weighting did not return usable results for the province of Nooristan.

Table S11: MCV1 status and proportion of children with probable MCV1 who also probably received MCV2 according to age at MCV1 for ADM1 level provinces of Afghanistan. The application of DHS survey data sampling weighting did not return usable results for the province of Nooristan. Cases where the number of children appears as equal to zero but the percentage and 95% confidence interval is non-zero are due to rounding effects after the application of weighting according to the DHS stratified sampling design. The application of DHS survey data sampling weighting did not return usable results for the province of Nooristan.

Region	Number of children with probable MCV1 by age 12 months	Number of children with probable MCV1 after age 12 months	Number (%, 95% CI) with probable MCV1 by 12 months whose MCV2 status was probable	Number (%, 95% CI) with probable MCV1 after age 12 months whose MCV2 status was probable	Right-tailed p-value for whether the proportion of probable MCV1 recipients who went on to receive MCV2 was greater for those who received MCV1 before age 12 months than for those who received MVC1 after age 12 months
Badakhshan	47	6	35 (74.7%, 57%-86.8%)	3 (54%, 16%-87.9%)	0.202
Badghis	36	14	22 (60.3%, 38.7%-78.5%)	7 (48.9%, 23.4%-75%)	0.267
Baghlan	41	5	30 (73.1%, 55.5%-85.5%)	3 (64.2%, 22.5%-91.7%)	0.35
Balkh	62	20	38 (60.8%, 42.9%-76.3%)	9 (45.3%, 19.6%-73.8%)	0.196
Bamyan	22	3	16 (72.4%, 56.9%-83.9%)	1 (31.6%, 8.7%-69%)	0.018
Daykundi	7	4	6 (73.7%, 44.9%-90.6%)	1 (24.1%, 5.6%-63.2%)	0.012
Farah	38	2	27 (69.6%, 52.5%-82.6%)	0 (0%, 0%-0%)	<0.0001
Faryab	154	16	82 (53.2%, 31.6%-73.7%)	9 (59.3%, 19.6%-89.7%)	0.404
Ghazni	34	6	28 (82%, 53.8%-94.7%)	3 (50%, 13.6%-86.4%)	0.115
Ghor	42	19	37 (87.6%, 72.3%-95%)	14 (74.9%, 48.7%-90.4%)	0.155
Helmand	13	5	11 (85.6%, 55.7%-96.6%)	4 (68.9%, 33.2%-90.8%)	0.201
Herat	125	8	96 (76.7%, 61.6%-87.1%)	3 (39.6%, 5.6%-87.9%)	0.113
Jawzjan	28	8	26 (91.7%, 77.7%-97.2%)	6 (70.2%, 42.6%-88.2%)	0.055
Kabul	194	133	161 (82.9%, 56%-94.9%)	102 (76.9%, 47.7%-92.4%)	0.348
Kandahar	58	33	21 (35.7%, 20.6%-54.4%)	13 (40.1%, 19.1%-65.6%)	0.391
Kapisa	6	3	5 (83.9%, 64.6%-93.7%)	2 (71%, 36.9%-91.1%)	0.226
Khost	24	9	21 (85.6%, 65%-95%)	3 (34.9%, 14%-63.8%)	0.001
Kunarha	21	8	17 (77.4%, 53.7%-91%)	2 (29.6%, 7.3%-69%)	0.013
Kunduz	70	3	22 (31.2%, 18.3%-47.8%)	2 (63.9%, 15.6%-94.5%)	0.124
Laghman	65	16	31 (46.7%, 34.6%-59.3%)	10 (59.4%, 33.6%-80.9%)	0.194
Logar	17	1	16 (93.1%, 77.8%-98.1%)	0 (41.5%, 13.5%-76.3%)	0.006
Nangarhar	55	15	43 (78.2%, 67.2%-86.3%)	9 (56.3%, 34.4%-76.1%)	0.041
Nimroz	12	2	9 (74.3%, 51.2%-88.8%)	1 (73.6%, 33.3%-93.9%)	0.486
Nooristan					
Paktika	59	3	20 (34.4%, 24.9%-45.4%)	1 (17.7%, 2.3%-66.2%)	0.169
Paktya	7	1	5 (72.5%, 42.9%-90.3%)	1 (79.1%, 34.4%-96.5%)	0.382
Panjsher	2	1	2 (87.7%, 71%-95.4%)	1 (84.1%, 39.3%-97.7%)	0.41
Parwan	36	13	33 (89.7%, 77%-95.8%)	10 (72.9%, 45.4%-89.7%)	0.097
Samangan	9	5	6 (64.2%, 38.2%-83.9%)	3 (51.1%, 21.9%-79.6%)	0.271
Sar-E-Pul	23	10	15 (63.9%, 42%-81.2%)	2 (18.1%, 6.1%-43%)	0.001
Takhar	75	9	57 (76.2%, 62.2%-86.1%)	3 (36.5%, 11.1%-72.6%)	0.022
Urozgan	0	0	0 (100%, 100%-100%)	0 (100%, 100%-100%)	
Wardak	33	5	23 (70.2%, 56.9%-80.7%)	1 (13.8%, 2.6%-48.7%)	<0.0001
Zabul	0	0	0 (100%, 100%-100%)	0 (0%, 0%-0%)	

Section 9

Table S12: Stratified results. Number, percentage and 95% CI of children with probable measles 1 vaccination whose measles 2 vaccination status was probable. Adjusted odds ratios, 95% CI and p-values for each of the stratification levels. Surveys for which MCV2 was introduced less than 3 years before the survey are denoted with an asterisk.

	Stratification variable	Strafication level	Number with probable MCV1 & Probable MCV2 / Number with probable MCV1 (%, 95% CI)	Adjusted Odds Ratio and 95% CI	p-value
Afghanistan 2015	Non-stratified		2517/3916 (64.3%, 61.5%-66.9%)		
	Sex	male	1311/2015 (65.0%, 61.3%-68.6%)		
		female	1207/1902 (63.5%, 59.4%-67.4%)	0.94 (0.75-1.17)	0.56
	Residency	urban	764/1014 (75.4%, 69.1%-80.7%)		
		rural	1753/2902 (60.4%, 57.5%-63.3%)	0.48 (0.27-0.87)	0.01
	Educational status of the mother	no education	1873/3068 (61.1%, 58.0%-64.0%)		
		primary	289/398 (72.6%, 64.3%-79.5%)	1.47 (0.98-2.22)	0.06
		secondary	271/350 (77.5%, 68.5%-84.4%)	1.99 (1.18-3.33)	0.01
		higher	84/100 (83.8%, 66.0%-93.2%)	2.54 (0.91-7.04)	0.07
	Wealth	poorest	447/787 (56.9%, 52.1%-61.5%)		
		poorer	445/761 (58.5%, 53.8%-63.0%)	1.1 (0.83-1.44)	0.52
		middle	431/705 (61.1%, 55.8%-66.2%)	1.17 (0.88-1.56)	0.28
		richer	528/724 (73.0%, 68.4%-77.1%)	1.78 (1.31-2.41)	<0.001
		richest	666/940 (70.8%, 61.9%-78.4%)	0.91 (0.46-1.79)	0.78
	Birth order	1	520/819 (63.4%, 56.4%-70.0%)		
		2-3	846/1239 (68.3%, 64.1%-72.2%)	1.34 (0.95-1.89)	0.09
		4-5	590/921 (64.1%, 58.0%-69.8%)	1.15 (0.78-1.67)	0.48
		6+	562/938 (59.9%, 54.8%-64.8%)	1.04 (0.72-1.49)	0.85

	Stratification variable	Strafication level	Number with probable MCV1 & Probable MCV2 / Number with probable MCV1 (%, 95% CI)	Adjusted Odds Ratio and 95% CI	p-value
**Angola 2015-16***	**Non-stratified**		660/1460 (45.2%, 41.5%-48.8%)		
	**Sex**	**male**	328/749 (43.8%, 38.8%-48.9%)		
		**female**	332/711 (46.7%, 41.4%-52.0%)	1.1 (0.82-1.48)	0.51
	**Residency**	**urban**	506/1087 (46.5%, 42.1%-51.1%)		
		**rural**	154/373 (41.1%, 35.6%-46.9%)	0.87 (0.56-1.35)	0.53
	**Educational status of the mother**	**no education**	125/286 (43.5%, 36.1%-51.3%)		
		**primary**	232/554 (41.8%, 36.2%-47.7%)	0.89 (0.59-1.33)	0.56
		**secondary**	274/568 (48.2%, 42.2%-54.4%)	0.98 (0.6-1.59)	0.94
		**higher**	29/52 (56.1%, 35.4%-75.0%)	1.08 (0.39-3.0)	0.88
	**Wealth**	**poorest**	80/185 (43.1%, 35.3%-51.2%)		
		**poorer**	99/248 (39.8%, 33.3%-46.7%)	0.82 (0.51-1.32)	0.42
		**middle**	162/352 (46.0%, 39.3%-52.9%)	0.98 (0.55-1.73)	0.94
		**richer**	155/366 (42.4%, 34.5%-50.7%)	0.81 (0.42-1.57)	0.54
		**richest**	164/309 (53.0%, 43.6%-62.2%)	1.21 (0.59-2.48)	0.6
	**Birth order**	**1**	151/314 (48.0%, 40.2%-55.9%)		
		**2-3**	245/501 (48.9%, 42.7%-55.1%)	1.02 (0.69-1.53)	0.91
		**4-5**	131/325 (40.2%, 33.0%-47.8%)	0.74 (0.47-1.17)	0.2
		**6+**	133/320 (41.7%, 34.0%-49.7%)	0.8 (0.5-1.27)	0.34

	Stratification variable	Strafication level	Number with probable MCV1 & Probable MCV2 / Number with probable MCV1 (%, 95% CI)	Adjusted Odds Ratio and 95% CI	p-value
**Bangladesh 2017-18**	**Non-stratified**		1400/1558 (89.9%, 88.0%-91.5%)		
	**Sex**	**male**	732/826 (88.6%, 85.8%-90.9%)		
		**female**	668/732 (91.3%, 88.7%-93.3%)	1.33 (0.91-1.95)	0.14
	**Residency**	**urban**	366/415 (88.2%, 84.3%-91.3%)		
		**rural**	1035/1144 (90.5%, 88.3%-92.2%)	1.33 (0.83-2.15)	0.24
	**Educational status of the mother**	**no education**	74/91 (81.0%, 70.6%-88.4%)		
		**primary**	363/412 (88.3%, 84.3%-91.4%)	1.64 (0.83-3.27)	0.16
		**secondary**	721/791 (91.2%, 88.6%-93.2%)	2.13 (1.05-4.32)	0.04
		**higher**	242/265 (91.4%, 86.4%-94.8%)	2.48 (1.01-6.11)	0.05
	**Wealth**	**poorest**	268/312 (86.1%, 81.4%-89.8%)		
		**poorer**	292/319 (91.5%, 87.6%-94.3%)	1.54 (0.87-2.73)	0.14
		**middle**	268/295 (90.8%, 86.2%-94.0%)	1.4 (0.78-2.51)	0.26
		**richer**	271/295 (91.9%, 87.6%-94.7%)	1.61 (0.87-2.95)	0.13
		**richest**	302/338 (89.2%, 84.3%-92.6%)	1.17 (0.6-2.27)	0.65
	**Birth order**	**1**	609/677 (89.8%, 86.8%-92.2%)		
		**2-3**	646/711 (90.9%, 88.2%-92.9%)	1.23 (0.8-1.9)	0.34
		**4-5**	121/142 (85.4%, 77.6%-90.9%)	0.85 (0.43-1.67)	0.63
		**6+**	24/28 (87.7%, 70.1%-95.6%)	1.28 (0.39-4.26)	0.68

	Stratification variable	Strafication level	Number with probable MCV1 & Probable MCV2 / Number with probable MCV1 (%, 95% CI)	Adjusted Odds Ratio and 95% CI	p-value
**Burundi 2016-17**	**Non-stratified**		1849/2336 (79.2%, 77.3%-80.9%)		
	**Sex**	**male**	938/1188 (78.9%, 76.3%-81.4%)		
		**female**	911/1148 (79.4%, 76.7%-81.8%)	1.02 (0.82-1.28)	0.83
	**Residency**	**urban**	180/238 (75.5%, 69.5%-80.7%)		
		**rural**	1669/2098 (79.6%, 77.6%-81.4%)	1.8 (1.15-2.8)	0.01
	**Educational status of the mother**	**no education**	827/1080 (76.6%, 73.6%-79.2%)		
		**primary**	803/990 (81.1%, 78.4%-83.6%)	1.3 (1.02-1.64)	0.03
		**secondary**	203/245 (82.8%, 77.0%-87.4%)	1.47 (0.92-2.35)	0.11
		**higher**	16/21 (78.0%, 55.4%-91.0%)	1.33 (0.43-4.13)	0.62
	**Wealth**	**poorest**	396/501 (79.0%, 74.8%-82.7%)		
		**poorer**	385/482 (79.9%, 75.7%-83.5%)	1.01 (0.72-1.42)	0.95
		**middle**	359/465 (77.2%, 72.7%-81.1%)	0.87 (0.62-1.22)	0.41
		**richer**	377/478 (78.9%, 74.7%-82.6%)	0.96 (0.68-1.35)	0.8
		**richest**	332/411 (81.0%, 76.5%-84.8%)	1.27 (0.77-2.08)	0.35
	**Birth order**	**1**	379/463 (81.9%, 77.8%-85.4%)		
		**2-3**	652/815 (80.0%, 76.9%-82.9%)	0.92 (0.66-1.27)	0.61
		**4-5**	419/548 (76.4%, 72.2%-80.1%)	0.75 (0.53-1.06)	0.11
		**6+**	399/510 (78.2%, 74.1%-81.9%)	0.85 (0.6-1.22)	0.39

	Stratification variable	Strafication level	Number with probable MCV1 & Probable MCV2 / Number with probable MCV1 (%, 95% CI)	Adjusted Odds Ratio and 95% CI	p-value
**Jordan 2017-18**	**Non-stratified**		1573/1693 (92.9%, 91.1%-94.4%)		
	**Sex**	**male**	785/843 (93.1%, 90.4%-95.1%)		
		**female**	787/849 (92.7%, 90.0%-94.7%)	0.96 (0.58-1.58)	0.88
	**Residency**	**urban**	1395/1497 (93.2%, 91.2%-94.8%)		
		**rural**	178/195 (90.9%, 87.0%-93.7%)	0.76 (0.47-1.23)	0.27
	**Educational status of the mother**	**no education**	17/18 (95.5%, 81.7%-99.0%)		
		**primary**	116/124 (92.9%, 86.6%-96.4%)	0.6 (0.11-3.4)	0.57
		**secondary**	827/892 (92.7%, 90.3%-94.6%)	0.55 (0.11-2.78)	0.47
		**higher**	613/658 (93.1%, 89.6%-95.5%)	0.52 (0.1-2.78)	0.45
	**Wealth**	**poorest**	414/451 (91.9%, 88.6%-94.3%)		
		**poorer**	361/393 (91.9%, 88.5%-94.4%)	1.08 (0.62-1.88)	0.78
		**middle**	340/363 (93.6%, 88.9%-96.4%)	1.38 (0.67-2.85)	0.38
		**richer**	296/315 (93.9%, 87.1%-97.2%)	1.45 (0.59-3.55)	0.42
		**richest**	162/172 (94.6%, 87.1%-97.9%)	1.58 (0.55-4.51)	0.39
	**Birth order**	**1**	378/403 (93.9%, 90.1%-96.3%)		
		**2-3**	690/752 (91.8%, 88.4%-94.3%)	0.73 (0.38-1.39)	0.34
		**4-5**	356/381 (93.7%, 90.4%-95.9%)	0.97 (0.48-1.96)	0.92
		**6+**	148/157 (93.9%, 89.7%-96.5%)	1.01 (0.44-2.32)	0.97

	Stratification variable	Strafication level	Number with probable MCV1 & Probable MCV2 / Number with probable MCV1 (%, 95% CI)	Adjusted Odds Ratio and 95% CI	p-value
**Malawi 2015-16***	**Non-stratified**		577/3043 (19.0%, 17.3%-20.8%)		
	**Sex**	**male**	282/1458 (19.4%, 17.0%-22.0%)		
		**female**	295/1585 (18.6%, 16.3%-21.2%)	0.96 (0.77-1.2)	0.72
	**Residency**	**urban**	99/401 (24.7%, 19.1%-31.4%)		
		**rural**	478/2642 (18.1%, 16.4%-19.9%)	0.74 (0.48-1.13)	0.16
	**Educational status of the mother**	**no education**	63/384 (16.5%, 12.6%-21.3%)		
		**primary**	383/2037 (18.8%, 16.8%-20.9%)	1.06 (0.74-1.53)	0.74
		**secondary**	118/570 (20.7%, 16.5%-25.5%)	1.04 (0.63-1.71)	0.88
		**higher**	13/51 (26.2%, 12.3%-47.3%)	1.22 (0.42-3.56)	0.71
	**Wealth**	**poorest**	120/701 (17.2%, 14.2%-20.6%)		
		**poorer**	133/726 (18.3%, 14.9%-22.2%)	1.05 (0.75-1.47)	0.77
		**middle**	106/583 (18.1%, 14.8%-22.1%)	1.06 (0.75-1.48)	0.75
		**richer**	111/544 (20.3%, 16.5%-24.8%)	1.15 (0.81-1.64)	0.42
		**richest**	108/488 (22.1%, 17.5%-27.5%)	1.11 (0.7-1.75)	0.67
	**Birth order**	**1**	162/763 (21.3%, 17.9%-25.2%)		
		**2-3**	195/1136 (17.1%, 14.7%-20.0%)	0.78 (0.58-1.04)	0.09
		**4-5**	172/720 (23.9%, 20.1%-28.1%)	1.21 (0.88-1.67)	0.25
		**6+**	49/424 (11.4%, 8.4%-15.3%)	0.52 (0.34-0.79)	<0.0001

	Stratification variable	Strafication level	Number with probable MCV1 & Probable MCV2 / Number with probable MCV1 (%, 95% CI)	Adjusted Odds Ratio and 95% CI	p-value
**Maldives 2016-17**	**Non-stratified**		386/469 (82.2%, 77.2%-86.3%)		
	**Sex**	**male**	192/236 (81.1%, 73.2%-87.1%)		
		**female**	194/233 (83.3%, 76.5%-88.3%)	1.38 (0.74-2.57)	0.32
	**Residency**	**urban**	131/159 (82.3%, 68.5%-90.9%)		
		**rural**	255/310 (82.1%, 77.9%-85.7%)	0.59 (0.13-2.76)	0.5
	**Educational status of the mother**	**no education**	2/4 (45.8%, 12.4%-83.5%)		
		**primary**	61/81 (76.2%, 62.6%-86.0%)	5.39 (0.64-45.29)	0.12
		**secondary**	226/271 (83.4%, 77.3%-88.1%)	6.12 (0.73-51.12)	0.09
		**higher**	97/114 (84.8%, 71.2%-92.7%)	6.83 (0.7-66.96)	0.1
	**Wealth**	**poorest**	92/108 (84.7%, 77.3%-90.1%)		
		**poorer**	84/104 (80.8%, 72.3%-87.2%)	0.75 (0.37-1.52)	0.42
		**middle**	86/103 (82.8%, 72.9%-89.5%)	0.69 (0.33-1.44)	0.32
		**richer**	66/78 (84.6%, 67.6%-93.5%)	0.6 (0.15-2.37)	0.47
		**richest**	59/76 (77.2%, 55.6%-90.2%)	0.26 (0.04-1.87)	0.18
	**Birth order**	**1**	174/193 (90.0%, 83.8%-94.0%)		
		**2-3**	177/229 (77.2%, 69.0%-83.8%)	0.36 (0.17-0.74)	0.01
		**4-5**	30/41 (73.9%, 54.2%-87.1%)	0.32 (0.1-1.03)	0.06
		**6+**	5/6 (77.9%, 39.1%-95.1%)	0.49 (0.09-2.61)	0.41

	Stratification variable	Strafication level	Number with probable MCV1 & Probable MCV2 / Number with probable MCV1 (%, 95% CI)	Adjusted Odds Ratio and 95% CI	p-value
Myanmar 2015-16	Non-stratified		486/658 (73.9%, 69.9%-77.6%)		
	Sex	male	232/324 (71.7%, 65.9%-77.0%)		
		female	254/334 (76.1%, 70.3%-81.1%)	1.31 (0.86-1.99)	0.2
	Residency	urban	128/156 (81.9%, 73.3%-88.2%)		
		rural	358/502 (71.5%, 66.8%-75.7%)	0.72 (0.39-1.34)	0.3
	Educational status of the mother	no education	63/92 (68.0%, 57.0%-77.3%)		
		primary	206/302 (68.3%, 61.8%-74.1%)	0.93 (0.53-1.65)	0.81
		secondary	164/200 (82.0%, 75.2%-87.3%)	1.85 (0.91-3.77)	0.09
		higher	54/64 (83.9%, 69.4%-92.2%)	2.17 (0.72-6.55)	0.17
	Wealth	poorest	127/187 (67.9%, 60.1%-74.8%)		
		poorer	91/127 (71.9%, 61.9%-80.1%)	1.12 (0.63-1.99)	0.7
		middle	90/120 (74.8%, 64.9%-82.7%)	1.2 (0.65-2.21)	0.57
		richer	108/132 (82.0%, 73.1%-88.4%)	1.48 (0.76-2.88)	0.25
		richest	70/92 (76.4%, 63.9%-85.6%)	0.76 (0.33-1.76)	0.53
	Birth order	1	198/253 (78.0%, 71.3%-83.5%)		
		2-3	194/271 (71.6%, 64.9%-77.5%)	0.85 (0.52-1.39)	0.52
		4-5	68/95 (71.8%, 60.9%-80.7%)	0.97 (0.49-1.89)	0.92
		6+	26/38 (68.8%, 53.8%-80.6%)	0.94 (0.43-2.05)	0.87

	Stratification variable	Strafication level	Number with probable MCV1 & Probable MCV2 / Number with probable MCV1 (%, 95% CI)	Adjusted Odds Ratio and 95% CI	p-value
**Nigeria 2018***	**Non-stratified**		908/3361 (27.0%, 25.3%-28.8%)		
	**Sex**	**male**	476/1703 (27.9%, 25.5%-30.5%)		
		**female**	432/1658 (26.1%, 23.7%-28.5%)	0.91 (0.76-1.09)	0.3
	**Residency**	**urban**	495/1674 (29.6%, 26.9%-32.4%)		
		**rural**	413/1687 (24.5%, 22.4%-26.7%)	0.94 (0.77-1.16)	0.57
	**Educational status of the mother**	**no education**	209/996 (21.0%, 18.2%-24.1%)		
		**primary**	140/498 (28.1%, 23.8%-32.8%)	1.42 (1.05-1.92)	0.02
		**secondary**	373/1347 (27.7%, 25.0%-30.5%)	1.34 (1.02-1.78)	0.04
		**higher**	186/519 (35.8%, 30.9%-41.1%)	1.97 (1.37-2.82)	<0.0001
	**Wealth**	**poorest**	107/460 (23.2%, 19.3%-27.7%)		
		**poorer**	118/578 (20.4%, 17.2%-23.9%)	0.78 (0.57-1.07)	0.12
		**middle**	192/684 (28.1%, 24.5%-32.1%)	1.07 (0.77-1.5)	0.68
		**richer**	228/780 (29.2%, 25.6%-33.1%)	1.05 (0.74-1.49)	0.77
		**richest**	263/859 (30.6%, 26.8%-34.7%)	0.96 (0.65-1.4)	0.82
	**Birth order**	**1**	201/720 (27.9%, 24.2%-32.0%)		
		**2-3**	348/1204 (28.9%, 26.0%-32.0%)	1.05 (0.83-1.34)	0.68
		**4-5**	194/785 (24.7%, 21.4%-28.5%)	0.9 (0.68-1.18)	0.43
		**6+**	165/652 (25.3%, 21.8%-29.2%)	1.05 (0.78-1.41)	0.75

	Stratification variable	Strafication level	Number with probable MCV1 & Probable MCV2 / Number with probable MCV1 (%, 95% CI)	Adjusted Odds Ratio and 95% CI	p-value
**Pakistan 2017-18**	**Non-stratified**		1279/1444 (88.5%, 86.2%-90.5%)		
	**Sex**	**male**	658/722 (91.1%, 88.2%-93.4%)		
		**female**	621/722 (86.0%, 82.2%-89.1%)	0.58 (0.37-0.9)	0.02
	**Residency**	**urban**	463/514 (90.0%, 86.5%-92.6%)		
		**rural**	816/930 (87.8%, 84.5%-90.4%)	1.15 (0.69-1.89)	0.6
	**Educational status of the mother**	**no education**	457/549 (83.3%, 78.8%-87.0%)		
		**primary**	285/319 (89.6%, 83.9%-93.4%)	1.59 (0.88-2.85)	0.12
		**secondary**	338/364 (92.8%, 88.4%-95.6%)	2.31 (1.21-4.4)	0.01
		**higher**	198/212 (93.4%, 88.3%-96.4%)	2.42 (1.09-5.37)	0.03
	**Wealth**	**poorest**	187/223 (83.9%, 76.7%-89.1%)		
		**poorer**	243/280 (86.8%, 80.8%-91.1%)	1.11 (0.58-2.11)	0.75
		**middle**	256/298 (85.9%, 79.3%-90.6%)	0.86 (0.43-1.73)	0.67
		**richer**	318/349 (91.2%, 86.3%-94.4%)	1.23 (0.59-2.6)	0.58
		**richest**	274/294 (93.4%, 89.2%-96.0%)	1.4 (0.57-3.44)	0.46
	**Birth order**	**1**	364/390 (93.4%, 89.3%-96.0%)		
		**2-3**	479/550 (87.1%, 82.9%-90.4%)	0.52 (0.28-0.97)	0.04
		**4-5**	313/353 (88.6%, 83.4%-92.4%)	0.68 (0.34-1.36)	0.27
		**6+**	123/152 (81.1%, 72.0%-87.7%)	0.47 (0.22-1.01)	0.05

	Stratification variable	Strafication level	Number with probable MCV1 & Probable MCV2 / Number with probable MCV1 (%, 95% CI)	Adjusted Odds Ratio and 95% CI	p-value
**Papua New Guinea 2016-18***	**Non-stratified**		838/1112 (75.4%, 71.7%-78.8%)		
	**Sex**	**male**	463/600 (77.3%, 72.3%-81.6%)		
		**female**	375/512 (73.2%, 67.5%-78.3%)	0.8 (0.55-1.17)	0.26
	**Residency**	**urban**	127/151 (84.3%, 78.3%-88.9%)		
		**rural**	711/961 (74.0%, 69.8%-77.8%)	0.77 (0.38-1.54)	0.46
	**Educational status of the mother**	**no education**	146/204 (71.5%, 61.6%-79.7%)		
		**primary**	422/567 (74.5%, 69.4%-78.9%)	1.06 (0.64-1.76)	0.81
		**secondary**	239/306 (77.9%, 70.1%-84.2%)	1.11 (0.61-2.0)	0.73
		**higher**	32/35 (91.5%, 80.9%-96.5%)	2.65 (0.85-8.27)	0.09
	**Wealth**	**poorest**	133/196 (67.7%, 57.4%-76.6%)		
		**poorer**	145/200 (72.6%, 63.3%-80.3%)	1.27 (0.68-2.37)	0.46
		**middle**	159/218 (72.8%, 64.0%-80.1%)	1.27 (0.7-2.31)	0.44
		**richer**	203/259 (78.2%, 71.9%-83.4%)	1.61 (0.92-2.82)	0.09
		**richest**	199/238 (83.4%, 73.9%-90.0%)	1.89 (0.81-4.42)	0.14
	**Birth order**	**1**	185/251 (73.8%, 64.3%-81.5%)		
		**2-3**	341/428 (79.7%, 74.5%-84.0%)	1.43 (0.82-2.47)	0.21
		**4-5**	209/293 (71.4%, 63.7%-78.0%)	0.95 (0.54-1.68)	0.86
		**6+**	103/140 (73.5%, 62.5%-82.2%)	1.13 (0.58-2.2)	0.71

	Stratification variable	Strafication level	Number with probable MCV1 & Probable MCV2 / Number with probable MCV1 (%, 95% CI)	Adjusted Odds Ratio and 95% CI	p-value
**Philippines 2017**	**Non-stratified**		858/1478 (58.1%, 54.6%-61.5%)		
	**Sex**	**male**	455/748 (60.8%, 55.9%-65.5%)		
		**female**	403/729 (55.3%, 50.2%-60.3%)	0.8 (0.6-1.07)	0.13
	**Residency**	**urban**	397/689 (57.7%, 51.8%-63.3%)		
		**rural**	461/789 (58.4%, 54.2%-62.5%)	1.14 (0.83-1.57)	0.41
	**Educational status of the mother**	**no education**	5/15 (34.0%, 12.5%-65.1%)		
		**primary**	125/227 (55.3%, 47.1%-63.3%)	2.47 (0.64-9.53)	0.19
		**secondary**	393/734 (53.6%, 48.6%-58.5%)	2.24 (0.59-8.47)	0.24
		**higher**	335/503 (66.6%, 60.4%-72.2%)	3.45 (0.89-13.37)	0.07
	**Wealth**	**poorest**	192/377 (50.9%, 44.6%-57.1%)		
		**poorer**	198/319 (62.0%, 55.3%-68.3%)	1.55 (1.05-2.29)	0.03
		**middle**	178/309 (57.5%, 49.3%-65.2%)	1.27 (0.81-1.98)	0.3
		**richer**	144/271 (53.0%, 44.5%-61.4%)	0.98 (0.6-1.59)	0.92
		**richest**	147/201 (73.1%, 63.0%-81.2%)	2.07 (1.13-3.79)	0.02
	**Birth order**	**1**	290/477 (60.8%, 54.3%-66.9%)		
		**2-3**	385/656 (58.7%, 53.5%-63.7%)	0.97 (0.69-1.35)	0.86
		**4-5**	121/237 (51.1%, 42.4%-59.8%)	0.78 (0.5-1.22)	0.28
		**6+**	62/107 (57.9%, 46.1%-68.8%)	1.11 (0.61-2.01)	0.73

	Stratification variable	Strafication level	Number with probable MCV1 & Probable MCV2 / Number with probable MCV1 (%, 95% CI)	Adjusted Odds Ratio and 95% CI	p-value
**Senegal 2017**	**Non-stratified**		1073/1892 (56.7%, 54.0%-59.4%)		
	**Sex**	**male**	545/962 (56.7%, 52.9%-60.3%)		
		**female**	527/930 (56.7%, 52.8%-60.5%)	0.99 (0.8-1.24)	0.94
	**Residency**	**urban**	426/754 (56.5%, 51.5%-61.4%)		
		**rural**	646/1138 (56.8%, 53.7%-59.8%)	1.32 (0.98-1.77)	0.07
	**Educational status of the mother**	**no education**	586/1064 (55.1%, 51.8%-58.3%)		
		**primary**	278/491 (56.7%, 50.8%-62.3%)	1.05 (0.79-1.4)	0.72
		**secondary**	168/276 (61.0%, 53.2%-68.2%)	1.27 (0.86-1.86)	0.23
		**higher**	40/62 (65.3%, 44.2%-81.7%)	1.69 (0.66-4.33)	0.28
	**Wealth**	**poorest**	210/400 (52.5%, 48.0%-57.1%)		
		**poorer**	230/410 (56.1%, 51.2%-61.0%)	1.15 (0.88-1.51)	0.31
		**middle**	223/395 (56.6%, 51.1%-61.9%)	1.24 (0.91-1.69)	0.17
		**richer**	196/314 (62.6%, 55.2%-69.5%)	1.69 (1.12-2.54)	0.01
		**richest**	212/374 (56.9%, 48.8%-64.6%)	1.26 (0.8-1.98)	0.33
	**Birth order**	**1**	253/430 (58.8%, 53.0%-64.4%)		
		**2-3**	368/649 (56.7%, 51.7%-61.5%)	0.95 (0.7-1.3)	0.76
		**4-5**	260/450 (57.8%, 52.4%-63.0%)	1.04 (0.74-1.45)	0.82
		**6+**	192/363 (52.8%, 47.3%-58.3%)	0.87 (0.61-1.22)	0.41

	Stratification variable	Strafication level	Number with probable MCV1 & Probable MCV2 / Number with probable MCV1 (%, 95% CI)	Adjusted Odds Ratio and 95% CI	p-value
**Senegal 2018**	**Non-stratified**		665/989 (67.2%, 63.5%-70.8%)		
	**Sex**	**male**	368/546 (67.4%, 62.3%-72.1%)		
		**female**	297/443 (67.0%, 61.3%-72.2%)	1.03 (0.75-1.42)	0.84
	**Residency**	**urban**	241/360 (66.9%, 59.0%-73.9%)		
		**rural**	424/629 (67.4%, 63.5%-71.1%)	2.05 (1.29-3.26)	<0.0001
	**Educational status of the mother**	**no education**	411/625 (65.7%, 61.2%-69.9%)		
		**primary**	123/197 (62.7%, 53.0%-71.6%)	0.8 (0.51-1.25)	0.33
		**secondary**	111/144 (77.0%, 67.8%-84.2%)	1.36 (0.79-2.37)	0.27
		**higher**	19/22 (85.9%, 54.7%-96.8%)	1.94 (0.35-10.72)	0.45
	**Wealth**	**poorest**	135/225 (60.2%, 53.7%-66.4%)		
		**poorer**	146/217 (67.5%, 60.7%-73.6%)	1.47 (0.97-2.22)	0.07
		**middle**	137/207 (66.1%, 58.2%-73.3%)	1.58 (1.02-2.45)	0.04
		**richer**	118/180 (65.6%, 55.2%-74.7%)	2.05 (1.14-3.68)	0.02
		**richest**	127/159 (80.0%, 67.5%-88.5%)	4.64 (2.04-10.53)	<0.0001
	**Birth order**	**1**	146/196 (74.7%, 66.7%-81.3%)		
		**2-3**	252/376 (66.9%, 60.5%-72.8%)	0.77 (0.47-1.24)	0.28
		**4-5**	143/235 (61.0%, 52.6%-68.8%)	0.63 (0.37-1.09)	0.1
		**6+**	123/182 (67.8%, 60.5%-74.3%)	0.9 (0.53-1.51)	0.68

	Stratification variable	Strafication level	Number with probable MCV1 & Probable MCV2 / Number with probable MCV1 (%, 95% CI)	Adjusted Odds Ratio and 95% CI	p-value
**Senegal 2019**	**Non-stratified**		705/1030 (68.4%, 64.6%-71.9%)		
	**Sex**	**male**	339/492 (69.0%, 63.5%-74.0%)		
		**female**	365/539 (67.8%, 62.6%-72.6%)	0.95 (0.68-1.33)	0.75
	**Residency**	**urban**	308/420 (73.4%, 66.2%-79.5%)		
		**rural**	396/611 (64.9%, 60.9%-68.8%)	1.04 (0.67-1.62)	0.86
	**Educational status of the mother**	**no education**	416/651 (63.9%, 59.3%-68.3%)		
		**primary**	162/219 (73.7%, 64.5%-81.2%)	1.36 (0.83-2.21)	0.22
		**secondary**	95/127 (75.2%, 64.9%-83.3%)	1.41 (0.79-2.49)	0.24
		**higher**	31/33 (94.6%, 82.5%-98.5%)	6.79 (1.65-27.89)	0.01
	**Wealth**	**poorest**	136/202 (67.3%, 60.8%-73.2%)		
		**poorer**	130/219 (59.2%, 52.5%-65.7%)	0.69 (0.46-1.02)	0.07
		**middle**	123/188 (65.7%, 57.3%-73.1%)	0.88 (0.55-1.41)	0.59
		**richer**	143/206 (69.2%, 59.6%-77.3%)	0.95 (0.55-1.65)	0.85
		**richest**	173/216 (80.2%, 69.0%-88.1%)	1.51 (0.72-3.16)	0.28
	**Birth order**	**1**	204/282 (72.5%, 64.6%-79.2%)		
		**2-3**	258/369 (70.0%, 63.5%-75.7%)	0.96 (0.6-1.54)	0.86
		**4-5**	143/215 (66.5%, 59.1%-73.2%)	0.9 (0.55-1.48)	0.68
		**6+**	99/165 (60.2%, 51.0%-68.6%)	0.73 (0.43-1.26)	0.26

	Stratification variable	Strafication level	Number with probable MCV1 & Probable MCV2 / Number with probable MCV1 (%, 95% CI)	Adjusted Odds Ratio and 95% CI	p-value
**Sierra Leone 2019**	**Non-stratified**		906/1369 (66.2%, 63.4%-68.8%)		
	**Sex**	**male**	474/694 (68.2%, 64.3%-71.9%)		
		**female**	432/675 (64.1%, 60.1%-67.8%)	0.84 (0.66-1.08)	0.17
	**Residency**	**urban**	332/500 (66.4%, 61.3%-71.1%)		
		**rural**	574/869 (66.0%, 62.7%-69.2%)	1.21 (0.82-1.8)	0.34
	**Educational status of the mother**	**no education**	455/712 (63.9%, 60.1%-67.6%)		
		**primary**	118/189 (62.6%, 55.1%-69.6%)	0.98 (0.69-1.4)	0.92
		**secondary**	307/433 (70.9%, 65.8%-75.4%)	1.48 (1.05-2.07)	0.03
		**higher**	26/36 (72.6%, 51.6%-86.8%)	1.58 (0.59-4.24)	0.36
	**Wealth**	**poorest**	192/291 (65.9%, 60.3%-71.1%)		
		**poorer**	195/301 (64.9%, 59.1%-70.2%)	0.92 (0.65-1.31)	0.66
		**middle**	191/292 (65.5%, 59.6%-71.0%)	0.95 (0.66-1.36)	0.77
		**richer**	173/263 (65.7%, 59.1%-71.8%)	1.05 (0.64-1.7)	0.86
		**richest**	155/223 (69.6%, 61.5%-76.6%)	1.14 (0.63-2.07)	0.67
	**Birth order**	**1**	210/321 (65.5%, 59.7%-71.0%)		
		**2-3**	362/531 (68.2%, 63.6%-72.3%)	1.2 (0.87-1.66)	0.27
		**4-5**	186/313 (59.5%, 53.6%-65.1%)	0.92 (0.63-1.34)	0.65
		**6+**	148/205 (72.2%, 65.5%-78.1%)	1.67 (1.08-2.59)	0.02

	Stratification variable	Strafication level	Number with probable MCV1 & Probable MCV2 / Number with probable MCV1 (%, 95% CI)	Adjusted Odds Ratio and 95% CI	p-value
**South Africa 2016**	**Non-stratified**		391/556 (70.4%, 65.4%-74.9%)		
	**Sex**	**male**	213/291 (73.3%, 66.3%-79.3%)		
		**female**	178/265 (67.2%, 59.8%-73.8%)	0.74 (0.47-1.18)	0.21
	**Residency**	**urban**	239/342 (70.0%, 62.7%-76.5%)		
		**rural**	152/214 (71.0%, 65.1%-76.2%)	1.67 (0.91-3.06)	0.1
	**Educational status of the mother**	**no education**	5/6 (83.1%, 49.3%-96.1%)		
		**primary**	31/51 (59.3%, 42.3%-74.3%)	0.26 (0.04-1.52)	0.13
		**secondary**	317/428 (74.0%, 68.6%-78.8%)	0.38 (0.08-1.96)	0.25
		**higher**	39/71 (55.4%, 39.7%-70.0%)	0.13 (0.02-0.76)	0.02
	**Wealth**	**poorest**	70/112 (62.6%, 52.9%-71.3%)		
		**poorer**	85/129 (66.0%, 55.4%-75.3%)	1.36 (0.72-2.54)	0.34
		**middle**	105/139 (75.1%, 64.8%-83.2%)	2.41 (1.14-5.11)	0.02
		**richer**	74/90 (81.9%, 70.4%-89.6%)	4.44 (1.68-11.76)	<0.0001
		**richest**	57/85 (67.2%, 51.2%-80.0%)	2.87 (1.04-7.91)	0.04
	**Birth order**	**1**	148/204 (72.5%, 64.6%-79.1%)		
		**2-3**	189/279 (67.9%, 60.3%-74.7%)	0.79 (0.48-1.29)	0.35
		**4-5**	44/53 (84.0%, 70.9%-91.9%)	2.62 (1.13-6.09)	0.03
		**6+**	9/19 (46.5%, 23.9%-70.6%)	0.42 (0.14-1.27)	0.12

	Stratification variable	Strafication level	Number with probable MCV1 & Probable MCV2 / Number with probable MCV1 (%, 95% CI)	Adjusted Odds Ratio and 95% CI	p-value
**Tanzania 2015-16***	**Non-stratified**		573/1639 (34.9%, 32.2%-37.8%)		
	**Sex**	**male**	311/863 (36.1%, 32.3%-40.1%)		
		**female**	261/777 (33.6%, 29.8%-37.7%)	0.87 (0.68-1.12)	0.28
	**Residency**	**urban**	179/451 (39.8%, 34.0%-45.8%)		
		**rural**	393/1188 (33.1%, 30.1%-36.3%)	0.96 (0.66-1.39)	0.83
	**Educational status of the mother**	**no education**	91/303 (30.0%, 24.3%-36.5%)		
		**primary**	361/1095 (32.9%, 29.7%-36.3%)	1.07 (0.76-1.5)	0.7
		**secondary**	110/225 (48.9%, 41.1%-56.8%)	2.07 (1.26-3.39)	<0.0001
		**higher**	11/17 (65.5%, 30.3%-89.2%)	3.86 (0.84-17.66)	0.08
	**Wealth**	**poorest**	97/351 (27.7%, 22.7%-33.3%)		
		**poorer**	112/350 (32.0%, 26.5%-38.0%)	1.23 (0.84-1.79)	0.29
		**middle**	129/356 (36.2%, 30.5%-42.4%)	1.39 (0.96-2.02)	0.08
		**richer**	115/292 (39.2%, 32.9%-45.9%)	1.48 (0.98-2.23)	0.06
		**richest**	120/290 (41.3%, 34.1%-48.9%)	1.24 (0.74-2.09)	0.41
	**Birth order**	**1**	137/393 (34.9%, 29.6%-40.6%)		
		**2-3**	227/580 (39.1%, 34.2%-44.3%)	1.33 (0.95-1.85)	0.09
		**4-5**	112/356 (31.5%, 26.2%-37.3%)	1.09 (0.75-1.58)	0.67
		**6+**	97/311 (31.1%, 25.5%-37.4%)	1.11 (0.75-1.65)	0.59

	Stratification variable	Strafication level	Number with probable MCV1 & Probable MCV2 / Number with probable MCV1 (%, 95% CI)	Adjusted Odds Ratio and 95% CI	p-value
**Zambia 2018**	**Non-stratified**		1188/1734 (68.5%, 65.6%-71.2%)		
	**Sex**	**male**	603/851 (70.9%, 67.2%-74.3%)		
		**female**	585/883 (66.2%, 61.8%-70.4%)	0.81 (0.63-1.05)	0.12
	**Residency**	**urban**	422/618 (68.3%, 62.4%-73.7%)		
		**rural**	766/1116 (68.6%, 65.5%-71.6%)	1.83 (1.15-2.91)	0.01
	**Educational status of the mother**	**no education**	96/166 (57.8%, 49.5%-65.6%)		
		**primary**	578/884 (65.4%, 61.4%-69.2%)	1.33 (0.91-1.94)	0.14
		**secondary**	465/629 (73.9%, 68.9%-78.4%)	2.06 (1.31-3.23)	<0.0001
		**higher**	49/55 (88.6%, 77.7%-94.6%)	4.72 (1.74-12.8)	<0.0001
	**Wealth**	**poorest**	255/420 (60.7%, 55.9%-65.3%)		
		**poorer**	249/350 (71.3%, 65.8%-76.2%)	1.53 (1.1-2.13)	0.01
		**middle**	232/333 (69.6%, 62.1%-76.2%)	1.56 (1.07-2.26)	0.02
		**richer**	242/361 (66.9%, 59.8%-73.3%)	1.65 (0.97-2.81)	0.07
		**richest**	210/270 (77.9%, 68.5%-85.2%)	2.73 (1.38-5.4)	<0.0001
	**Birth order**	**1**	306/423 (72.3%, 67.1%-76.9%)		
		**2-3**	405/612 (66.1%, 60.5%-71.3%)	0.76 (0.54-1.08)	0.12
		**4-5**	274/385 (71.1%, 65.4%-76.1%)	1.13 (0.78-1.64)	0.52
		**6+**	204/314 (64.9%, 58.4%-70.8%)	0.88 (0.59-1.3)	0.52

Section 10

Table S13: List of all DHS surveys for which survey files were downloaded and of 19 surveys with useful data. Surveys for which MCV2 was introduced less than 3 years before the survey are denoted with an asterisk.

Filenames as downloaded from DHS	surveys that contained relevant data	Country and year of survey as listed in the .FRQ file of the downloaded data and in https://dhsprogram.com/data/available-datasets.cfm	Year of introduction of MCV2 in all of the country (year in parenthesis is introduction in part of the country) from https://immunizationdata.who.int/pages/vaccine-intro-by-antigen/mcv2.html
AFKR71DT	X	Afghanistan 2015	2004
ALKR51DT			
ALKR71DT			
AOKR71DT	X	Angola 2015-16*	2015
AMKR42DT			
AMKR54DT			
AMKR61DT			
AMKR72DT			
AZKR52DT			
BDKR31DT			
BDKR3ADT			
BDKR41DT			
BDKR4JDT			
BDKR51DT			
BDKR61DT			
BDKR72DT			
BDKR7RDT	X	Bangladesh 2017-18	2012
BJKR31DT			
BJKR41DT			
BJKR51DT			
BJKR61DT			
BJKR71DT			
BOKR01DT			
BOKR31DT			
BOKR3BDT			
BOKR41DT			
BOKR51DT			
BRKR01DT			
BRKR21DT			
BRKR31DT			
BFKR21DT			
BFKR31DT			

BFKR43DT			
BFKR62DT			
BUKR01DT			
BUKR61DT			
BUKR71DT	X	Burundi 2016-17	2013
KHKR42DT			
KHKR51DT			
KHKR61DT			
KHKR73DT			
CMKR21DT			
CMKR31DT			
CMKR44DT			
CMKR61DT			
CMKR71DT			
CFKR31DT			
TDKR31DT			
TDKR41DT			
TDKR71DT			
COKR01DT			
COKR22DT			
COKR31DT			
COKR41DT			
COKR53DT			
COKR61DT			
COKR72DT			
KMKR31DT			
KMKR61DT			
CGKR51DT			
CGKR61DT			
CDKR51DT			
CDKR61DT			
CIKR35DT			
CIKR3ADT			
CIKR62DT			
DRKR01DT			
DRKR21DT			
DRKR31DT			
DRKR41DT			
DRKR4BDT			
DRKR52DT			
DRKR5ADT			
DRKR61DT			
DRKR6ADT			

ECKR01DT			
EGKR01DT			
EGKR21DT			
EGKR33DT			
EGKR42DT			
EGKR4ADT			
EGKR51DT			
EGKR5ADT			
EGKR61DT			
SZKR51DT			
ETKR41DT			
ETKR51DT			
ETKR61DT			
ETKR71DT			
GAKR41DT			
GAKR61DT			
GMKR61DT			
GHKR01DT			
GHKR31DT			
GHKR41DT			
GHKR4BDT			
GHKR5ADT			
GHKR72DT			
GUKR01DT			
GUKR34DT			
GUKR41DT			
GUKR71DT			
GNKR41DT			
GNKR52DT			
GNKR62DT			
GNKR71DT			
GYKR5IDT			
HTKR31DT			
HTKR42DT			
HTKR52DT			
HTKR61DT			
HTKR71DT			
HNKR52DT			
HNKR62DT			
APKR42DT			
ARKR42DT			
ASKR42DT			
BHKR42DT			

DLKR42DT			
GJKR42DT			
GOKR42DT			
HPKR42DT			
HRKR42DT			
IAKR23DT			
IAKR42DT			
IAKR52DT			
IAKR74DT			
JMKR42DT			
KAKR42DT			
MGKR42DT			
MHKR42DT			
MNKR42DT			
MPKR42DT			
MZKR42DT			
NAKR42DT			
ORKR42DT			
PJKR42DT			
RJKR42DT			
SKKR42DT			
TNKR42DT			
TRKR42DT			
UPKR42DT			
WBKR42DT			
IDKR01DT			
IDKR21DT			
IDKR31DT			
IDKR3ADT			
IDKR42DT			
IDKR51DT			
IDKR63DT			
IDKR71DT			
JOKR21DT			
JOKR31DT			
JOKR42DT			
JOKR51DT			
JOKR61DT			
JOKR6CDT			
JOKR73DT	X	Jordan 2017-18	1995
KKKR31DT			
KKKR42DT			
KEKR01DT			

KEKR31DT			
KEKR3ADT			
KEKR42DT			
KEKR52DT			
KEKR72DT			
KYKR31DT			
KYKR61DT			
LSKR41DT			
LSKR61DT			
LSKR71DT			
LBKR01DT			
LBKR51DT			
LBKR6ADT			
LBKR7ADT			
MDKR21DT			
MDKR31DT			
MDKR42DT			
MDKR51DT			
MWKR21DT			
MWKR41DT			
MWKR4EDT			
MWKR61DT			
MWKR7ADT	X	Malawi 2015-16*	2015
MVKR52DT			
MVKR71DT	X	Maldives 2016-17	2007
MLKR01DT			
MLKR31DT			
MLKR41DT			
MLKR53DT			
MLKR6ADT			
MLKR7ADT			
MXKR01DT			
MBKR53DT			
MAKR01DT			
MAKR21DT			
MAKR43DT			
MZKR31DT			
MZKR41DT			
MZKR62DT			
MMKR71DT	X	Myanmar 2015-16	2012 (2008)
NMKR21DT			
NMKR41DT			
NMKR51DT			

NMKR61DT			
NPKR31DT			
NPKR41DT			
NPKR51DT			
NPKR61DT			
NPKR7HDT			
NCKR31DT			
NCKR41DT			
NIKR21DT			
NIKR31DT			
NIKR51DT			
NIKR61DT			
NGKR21DT			
NGKR4BDT			
NGKR53DT			
NGKR6ADT			
NGKR7ADT	X	Nigeria 2018*	2020 (2019)
OSKR01DT			
PKKR21DT			
PKKR52DT			
PKKR61DT			
PKKR71DT	X	Pakistan 2017-18	2009
PGKR71DT	X	Papua New Guinea 2016-18*	2016
PYKR21DT			
PEKR01DT			
PEKR21DT			
PEKR31DT			
PEKR41DT			
PEKR51DT			
PEKR5ADT			
PEKR5IDT			
PEKR61DT			
PEKR6ADT			
PEKR6IDT			
PHKR31DT			
PHKR3BDT			
PHKR41DT			
PHKR52DT			
PHKR61DT			
PHKR71DT	X	Philippines 2017	2010 (2009)
RWKR21DT			
RWKR41DT			
RWKR53DT			

RWKR5ADT			
RWKR61DT			
RWKR70DT			
STKR51DT			
SNKR01DT			
SNKR21DT			
SNKR32DT			
SNKR4ADT			
SNKR61DT			
SNKR6DDT			
SNKR70DT			
SNKR7HDT			
SNKR7IDT			
SNKR7ZDT	X	Senegal 2017	2014
SNKR81DT	X	Senegal 2018	2014
SNKR8BDT	X	Senegal 2019	2014
SLKR51DT			
SLKR61DT			
SLKR7ADT	X	Sierra Leone 2019	2015
ZAKR31DT			
ZAKR71DT	X	South Africa 2016	1994
LKKR01DT			
SDKR01DT			
TJKR61DT			
TJKR71DT			
TZKR21DT			
TZKR3ADT			
TZKR41DT			
TZKR4IDT			
TZKR63DT			
TZKR7BDT	X	Tanzania 2015-16*	2014
THKR01DT			
TLKR61DT			
TLKR71DT			
TGKR01DT			
TGKR31DT			
TGKR61DT			
TTKR01DT			
TNKR01DT			
TRKR31DT			
TRKR41DT			
TRKR4ADT			
TRKR51DT			

TRKR62DT			
UGKR01DT			
UGKR33DT			
UGKR41DT			
UGKR52DT			
UGKR61DT			
UGKR7BDT			
UAKR51DT			
UZKR31DT			
VNKR31DT			
VNKR41DT			
YEKR21DT			
YEKR61DT			
ZMKR21DT			
ZMKR31DT			
ZMKR42DT			
ZMKR51DT			
ZMKR61DT			
ZMKR71DT	X	Zambia 2018	2013
ZWKR01DT			
ZWKR31DT			
ZWKR42DT			
ZWKR52DT			
ZWKR62DT			
ZWKR72DT			

References

1 WHO. *Vaccination schedule for Measles*, <https://immunizationdata.who.int/pages/schedule-by-disease/measles.html?ISO_3_CODE=AFG+AGO+BGD+BDI+JOR+MMR+MDV+MWI+NGA+PNG+PHL+PAK+SLE+SEN+TZA+ZAF+ZMB&TARGETPOP_GENERAL=>.

2 WHO. *WHO/UNICEF Joint Reporting Process*, <https://www.who.int/teams/immunization-vaccines-and-biologicals/immunization-analysis-and-insights/global-monitoring/who-unicef-joint-reporting-process>.
